# CCT196969 inhibits TNBC by targeting the HDAC5/RXRA/ASNS axis to down-regulate asparagine synthesis

**DOI:** 10.1186/s13046-025-03494-5

**Published:** 2025-08-08

**Authors:** Qiong Yuan, Qi Wang, Jun Li, Liyang Yin, Shu Liu, Xuyu Zu, Yingying Shen

**Affiliations:** Cancer Research Institute, The First Affiliated Hospital, Hengyang Medical School, University of South China, Hengyang, Hunan 421001 PR China

**Keywords:** TNBC, CCT196969, Asparagine, HDAC5, RXRA

## Abstract

**Background:**

Triple-negative breast cancer (TNBC) seriously threatens the health of patients, and new therapeutic targets and drugs need to be explored. Studies have shown that CCT196969 can inhibit melanoma and colorectal cancer. However, the role of CCT196969 in TNBC is unclear.

**Methods:**

CCT196969 inhibited TNBC via in vitro and in vivo experiments. Transcriptomic analysis, metabolomics analysis, proteomic analysis, and other experiments were used to determine that CCT196969 inhibited asparagine synthetase (ASNS) expression and downstream mTOR signaling pathway, and that Retinoid X Receptor Alpha (RXRA) was the upstream transcription factor that regulated ASNS. The binding sites of RXRA and ASNS promoter were determined by luciferase and Chromatin Immunoprecipitation (CHIP) assay. Histone Deacetylase 5 (HDAC5) was confirmed as the key target of CCT196969 by target capture assay, Cell thermal shift assay (CETSA), Surface plasmon resonance (SPR) and other experiments. qPCR, CHX tracer, MG132, immunofluorescence (IF) and Co-Immunoprecipitation (CO-IP) assay were used to detect the regulation of HDAC5 on RXRA transcription and post-translation level, and the key domains of interaction and binding between HDAC5 and RXRA. The binding sites of HDAC5 and RXRA were predicted by PyMOL software. The effect of HDAC5 on the acetylation and ubiquitination levels of RXRA was examined by CO-IP experiment. The deacetylation site of HDAC5 to RXRA was investigated by IP experiments and mass spectrometry.

**Results:**

This study reveals that CCT196969 can inhibit TNBC by down-regulating the expression of ASNS, inhibiting asparagine synthesis and downstream mTORC pathway. Mechanistically, CCT196969 targeted and inhibited HDAC5, reducing the interaction of its 1-291 region with RXRA’s 1–98 region, and further resulting in an increase in RXRA acetylation (K410 and K412) and a decrease in ubiquitination levels. Together, these effects up-regulated the transcriptional and post-translational levels of RXRA. Finally, RXRA inhibited the expression of ASNS at the transcriptional level by binding to the − 1114/-1104 region on the ASNS promoter as a transcription suppressor.

**Conclusions:**

This study reveals a previously unrecognized anti-TNBC mechanism of CCT196969 through the HDAC5/RXRA/ASNS axis. This provides potential candidate targets for the treatment of TNBC and a theoretical basis for the clinical treatment of TNBC patients with CCT196969.

**Supplementary Information:**

The online version contains supplementary material available at 10.1186/s13046-025-03494-5.

## Introduction

Breast cancer, the most prevalent cancer among women, can develop distant metastases in advanced stages, resulting in multiple organ lesions that pose a significant threat to life. TNBC constitutes 10–20% of breast cancer cases and is recognized as the most heterogeneous and aggressive subtype, associated with a poor prognosis [1]. TNBC is characterized by the absence of estrogen receptors (ER), progesterone receptors (PR), and human epidermal growth factor receptor 2 (HER2), rendering it insensitive to endocrine and HER2-targeted therapies [2]. Systemic chemotherapy has traditionally been the primary treatment for TNBC; however, it often results in severe side effects and lacks an effective, low-toxicity treatment plan [3, 4]. Research indicates that metabolic reprogramming of tumor cells is a crucial mechanism enabling their adaptation to adverse environments, as well as their rapid proliferation, invasion, and metastasis [5]. This insight may offer new strategies and potential targets for cancer treatment.

Asparagine, a non-essential amino acid, plays an important role in the growth and metastasis of tumor cells. It is involved in nutrient metabolism of tumor cells by activating mTORC signaling pathway and promoting tumor metastasis through its influence on the epithelial-mesenchymal transition (EMT) pathway [6, 7]. ASNS utilizes the amino group provided by glutamine to convert aspartate into asparagine for use by cells. When the synthesis of asparagine is inhibited, it will hinder tumor growth and metastasis, and loss of ASNS expression will further increase the sensitivity of cells to asparagine depletion [6, 8]. ASNase reduces asparagine levels by hydrolyzing asparagine, thereby inhibiting the growth of leukemia cells. However, the therapeutic efficacy of ASNase in solid tumors is limited, because tumor cells can upregulate ASNS to fulfill their requirements through metabolic reprogramming [9]. Evidence suggests that knockdown of ASNS can inhibit cancer cell growth, highlighting its potential as a therapeutic target [10].

CCT196969, a small molecule compound, is an orally active pan-RAF inhibitor that also targets SRC-family kinases (SFK). It exhibits potential inhibitory activity against melanoma and colorectal cancer cell lines with BRAF mutations, particularly those harboring BRAF and NRAS mutations. This provides a promising treatment option for patients who have developed resistance to first-line inhibitors targeting BRAF and MEK. Furthermore, recent evidence indicates that CCT196969 can inhibit the proliferation, invasion, and migration of melanoma brain metastatic cells in vitro [11, 12]. However, the role of CCT196969 in breast cancer, particularly TNBC, remains unclear.

Our study found that CCT196969 significantly inhibited the proliferation, invasion, and migration of TNBC cell lines both in vitro and in vivo. This effect is mediated through the down-regulation of ASNS expression, which further inhibits asparagine synthesis and its downstream mTOR signaling pathway. Mechanistically, CCT196969 targets HDAC5 in TNBC cells, rather than its common targets BRAF, RAF1, and SRC, thereby inhibiting ASNS expression via the HDAC5/RXRA/ASNS axis. This study identifies potential therapeutic targets for TNBC and provides a theoretical basis for the clinical application of CCT196969 in the treatment of triple-negative breast cancer.

## Materials and methods

### Cell culture, transfection and infection

The cell lines used in this study were cultured in a constant temperature incubator containing 5% CO2 at 37℃ and grew well in DMEM medium (#11965092, Gibco) containing 10% FBS (#A5670701, Gibco) and 1% penicillin/streptomycin (#P1400, Solarbio). Before transfection, cells were seeded in cell culture plates and the complete medium was replaced with Opti-MEM medium (#31985070, Gibco) after cell adhesion. Lipofectamine 3000 reagent (#L3000001, Thermo Fisher Scientific) was mixed with plasmid/siRNA in Opti-MEM according to the manufacturer’s instructions and allowed to stand for 20 min before being added to cells. For stably expressed cell lines, the required virus volume was calculated based on the MOI value, and then the target virus was added to the cells for infection. The sequence information of siRNA and shRNA used is shown in Supplementary Table 1.

### Cell proliferation experiment

Cells from different treatment groups were digested into cell suspension, counted and then planted on cell culture plates. 100 µL suspensions containing 1000 cells were planted in 96-well plates (#701101, NEST), and each group was cultured at different time points (0 h, 24 h, 48 h, 72 h, 96 h), with 3 repeat wells at each time point. Subsequently, 10 µL CCK-8 reagent (#BMU106-CN, Abbkine) was added at the corresponding time point of culture, and the absorbance at 490 nm was measured by enzyme-label after incubation in the cell incubator for 2 h. In addition, cells (1000/well) were planted on 6-well plates (#703001, NEST) for plate cloning formation experiment, each group was repeated 3 times, and the cell colonies were stained with 10% crystal violet (#ICO600, Solarbio).

### Transwell

40 µL Matrigel solution (#354248, Corning) was added to the upper chamber of Transwell plate (#3422, Corning) and solidified for 30 min at 37℃ (Matrigel was not required for migration experiments). The treated cells were mixed with DMEM and counted, and planted in the upper compartment (5 × 10⁴/well for migration experiment and 1 × 10⁵/well for invasion experiment). The upper compartment was supplemented with serum-free medium to 300 µL, and the lower compartment with 500 µL complete medium as chemokines. Cells were cultured for 48 h in the incubator, stained with 10% crystal violet, and the cells that did not penetrate the membrane were wiped off with a cotton swab. The cells that did penetrate the membrane were observed in 5 fields randomly selected under the microscope and counted.

### Cell apoptosis assay

The cells before and after CCT196969 treatment were collected and mixed with 100 µL binding buffer. Then 5 µL FITC and 5 µL PI staining solution were added to the mixture and incubated for 5 min at room temperature in the dark. 400 µL of binding buffer was added to each tube, mixed and tested on the machine. All the reagents are from apoptosis detection kits (#KTA0005, Abbkine).

### Western blot

The cells were collected in a 1.5 mL centrifuge tube and added with RIPA lysate (#P0013B, Beyotime Biotechnology) containing protease inhibitor (#P1005, Beyotime Biotechnology), cracked on ice for 30 min, centrifuged at 14,000 rpm for 10 min, the supernatant was quantified by BCA method (#CW0014S, Cowin Biotech), then protein loading buffer was added, and the protein was denatured by heating at 95 °C for 10 min. Proteins were subjected to SDS-PAGE and transferred to PVDF membranes (#IPFL00010, Merck Millipore), blocked with 10% milk (#A600669, Sangon) for 2–3 h, and incubated with primary antibodies overnight at 4 °C. The next day, the protein membrane was washed 3 times with TBST (#G0001, Servicebio) and incubated in the secondary antibody at room temperature for 1 h. Subsequently, the membrane was washed with TBST for 3 times and developed with ECL luminescent solution (#E423, Vazyme). All membranes were cut before antibody incubation.

### qPCR

Total RNA was extracted with TRIzol reagent (#15596018CN, Thermo Fisher Scientific), and cDNA was synthesized with HiScript Ill All-in-one RT SuperMix (#R323, Vazyme) from 1 µg RNA. Real-time quantitative PCR was then performed in the Roche LightCycler^®^ 480 system using SYBR Premix DimerEraser (#RR091A, Takara) and specific primers of related genes. Primer sequences for qPCR are listed in Supplementary Table 2.

### Luciferase assay

The ASNS promoter was cloned into the PGL3 vector, and the bidirectional primer sequence was designed to mutate the predicted site of RXRA binding to the ASNS promoter. Subsequently, the plasmids were transformed, and a single colony was selected and sent to GENEWIZ for sequencing. Primers for generating plasmid constructs and primers for plasmid sequencing are listed in Supplementary Tables 3, 4. The mutant plasmid was cultured and extracted according to the instructions of Jiangsu Cowin Biotech Co., Ltd (#CW2108M). The mutant plasmid was transfected into the cells using Lipofectamine 3000 reagent (#L3000001, Thermo Fisher Scientific). After 48 h, the cells were collected and lysed according to the Dual Luciferase Reporter Gene Assay Kit (#RG088S, Beyotime Biotechnology). The RLU values of firefly and renilla were recorded and the ratio was calculated for analysis.

### CHIP

4T1 cells treated/untreated with CCT196969 were collected and chromatin immunoprecipitation was performed according to the ab500 CHIP kit (Abcam). In brief, proteins were cross-linked to DNA with 1.1% formaldehyde, followed by quenching the formaldehyde with glycine and washing with PBS. The cells were cleaved with CHIP lysate, treated with ultrasound and supernatant was obtained by centrifugation. The Input group was retained, and the remaining samples were equally divided, anti-RXRA antibody and anti-IgG antibody were added, respectively, and rotated overnight at 4 °C. The next day, agarose beads were added and spun at 4 °C for 2 h. Subsequently, beads were washed and mixed with DNA purifying slurry, incubated at 98 °C for 10 min, placed at room temperature for 20 min, and proteinase K was added to obtain DNA fragments. The following primers were designed: ASNS-mut4-F: CTGCCTACAGCTCTCTGAA; ASNS-mut4-R: TGTCCTAAGAAACTGTTGTGC. Finally, qPCR was performed on the extracted DNA and the results were expressed as Fold Enrichment.

### CO-IP

The CDS sequences of HDAC5 and RXRA were cloned into pcDNA3.1 vector and labeled with HA and FLAG tags, respectively. The amino acid truncated fragments of HDAC5 and RXRA were constructed by homologous recombination. Primers for generating amino acid snippets and primers for sequencing the plasmid are listed in Supplementary Table 5. The plasmid was transfected with Lipofectamine 3000 (#L3000001, Thermo Fisher Scientific), cells were collected 48 h later, the cells were lysed with IP lysate (#P0013, Beyotime) and ultrasounded, and the supernatant was obtained by centrifugation at 14,000 rpm for 10 min. The protein was quantified by BCA method, anti-HA or anti-FLAG antibody was added, and the protein was rotated overnight at 4℃. On the second day, magnetic agarose beads (#B23202, Bimake) were added and rotated at 4℃ for 6–8 h. The beads were washed with IP cracking solution on a magnetic separator, and then 2× loading buffer was added and heated at 95℃ for 5 min. Finally, the enriched proteins were analyzed by Western Blot.

### IF

Cell climbing glass coverslips were prepared, fixed with 4% paraformaldehyde for 20 min, washed with PBS (#G4202, Servicebio) and permeated with 0.5% TritonX-100 (#85111, Thermo Fisher Scientific) for 15–20 min, and then closed with 3% BSA (#ST023, Beyotime) for 30 min. Antibody was added and incubated at 4℃ overnight. On the second day, fluorescein labeled antibodies were added and incubated for 1 h at room temperature and away from light. Then the cells were washed with PBS and incubated for 3–5 min with the anti-fluorescence quencher containing DAPI (#abs47047616, Absin). Observation was made under a fluorescence microscope and photos were taken.

### CETSA

After 4T1/ MDA-MB-231/HCC-1806 cells were treated with CCT196969 for 4 h, the cells were mixed with PBS containing protease inhibitors. Cell suspension was evenly divided into 5 tubes (40 µl/tube), heated at 42℃, 45℃, 48℃, 51℃, 54℃ for 3 min, and cooled at room temperature for 3 min, respectively. Then the cells were repeatedly freeze-thawed in liquid nitrogen for 3–4 times, and the supernatant was obtained by centrifugation at 4℃ and 13,000 rpm for 40 min. After protein quantification, sample buffer was added, heated at 99℃ for 15 min, cooled at 4℃, and Western Blot assay was performed.

### SPR

CCT196969 was printed onto the microarray via SPRi-arrayer. The protein concentration of HDAC5 was adjusted. The affinity constants between CCT196969 and HDAC5 at different concentrations were determined by the SPR measurement method, and the signal curves were output (performed in Better Ways, Guangzhou).

### Mouse studies

Female Balb/c mice aged 6–8 weeks were selected, 4T1 cells (2 × 10^6^/100µL) injected into the fourth pair of mammary subcutaneous fat pads. After the tumor volume reached 80-100mm^3^, the mice were randomly divided into experimental group and control group. For single-drug treatment experiment, the experimental group were treated with 10 mg/kg CCT196969 every day. For combined treatment experiment, the experimental group was given 5 mg/kg CCT196969 or 40UI ASNase (MCE, HY-P1923) every day, the combined group was given 5 mg/kg CCT196969 and 40UI ASNase every day, and the control group was given the same amount of sterile water. The changes of weight and tumor volume of mice were observed. The experiment was terminated when the tumor of the control group reached a certain volume. The mice were killed and the tumor was removed, photographed and weighed, and stored at -80℃ for Western Blot detection.

### Mouse lung metastasis model

Female BALB/c mice about 4–5 weeks old were selected and 5 × 10^5^ 4T1-luc cells were injected into the mice through the tail vein. Mice were randomly grouped. From the first day after tumor inoculation, mice were gavaged with 10 mg/kg CCT196969 or Vehicle every day. The body weight of mice was continuously recorded during the experiment. After treating the mice with CCT196969 for 11 days, in vivo imaging of the mice was performed using the IVIS Spectrum system. Meanwhile, the lung tissues of mice were taken by lung perfusion method, fixed and stained with HE, and the number and area of the tumor nodules were counted under a microscope.

### Bioinformatics analyses

Transcription factors that could bind to the ASNS promoter was predicted by using the Database of Human Transciption Factor Targets (http://bioinfo.life.hust.edu.cn/hTFtarget#!/target). The promoter sequence information of mouse ASNS was searched by using NCBI website (https://www.ncbi.nlm.nih.gov/). The binding site of the mouse transcription factor RXRA to the ASNS promoter was predicted by using the JASPAR website (https://jaspar.elixir.no/). HDAC5 and RXRA functional domains was searched by using The UniProt website (https://www.uniprot.org/). The protein-protein binding sites was predicted by using ‌AlphaFold3. PyMOL visualizes protein-protein docking. Gene expression and correlation analysis by using TCGA (https://www.cancer.gov/aboutnci/organization/ccg/research/structural-genomics/tcga) and tissue chip (AF-BrcSur2201, AiFang biological). Overall survival (OS) was predicted by using the Kaplan-Meier Plotter website (https://kmplot.com/analysis/index.php?p=service).

## Result

### Small molecule compound CCT1969696 effectively inhibits TNBC

**Fig. 1:**
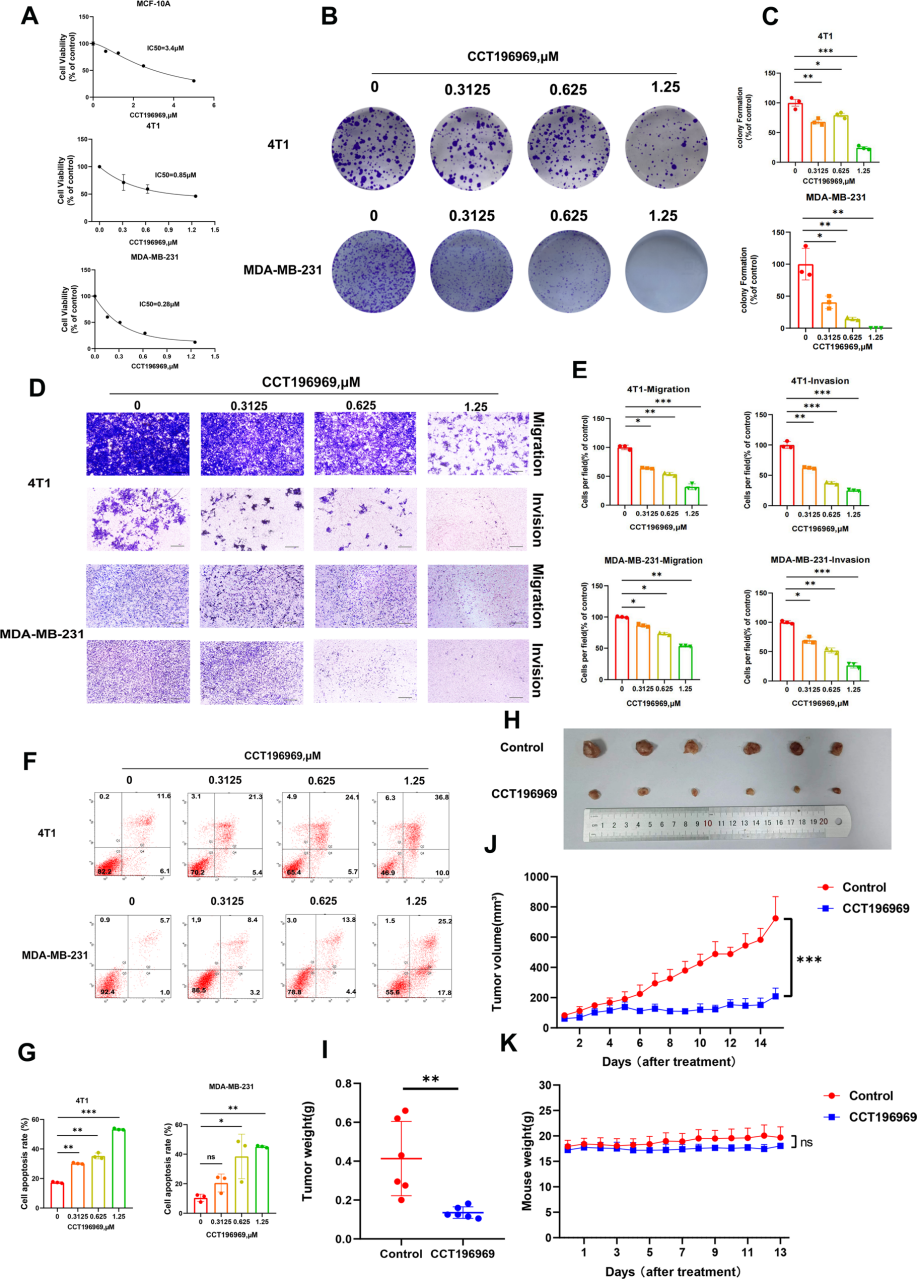
CCT196969 significantly inhibits TNBC in vitro and in vivo. **A**-**G**. 4T1 cells and MDA-MB-231 cells were treated with different concentrations of CCT196969 for 48 h, and the following experiments were performed: **A**. CCK-8 experiment, with data from three repeated experiments. **B**-**C**. Clone formation experiment was repeated three times, the number of cell colonies was counted, and the effect of CCT196969 on cell growth was observed. Figure **B** is the representative result, and Figure C is the statistical figure. **D**-**E**. Cell invasion and migration experiment, three independent data were collected for statistical analysis. Figure **D** is the typical result, and Figure **E** is the statistical figure. Scale bars, 20 μm. **F**-**G**. Apoptosis experiment, Annexin V-FITC/PI double staining flow assay was used to collect three independent data for statistical analysis. Figure **F** represents the typical result and Figure G is the statistical graph. **H**. Comparison of tumor tissue size between Control group and CCT196969 group (10 mg/kg, gavage once a day), where *n* = 6 in Control group and *n* = 6 in CCT196969 group. **I**. Tumor weight statistics of mice in Control group and CCT196969 treatment group. **J**. Tumor growth curves of mice in Control group and CCT196969 treatment group. **K**. Weight changes of mice in Control group and CCT196969 treatment group. All values are mean ± SD. The statistical significance was determined by t test, *, *P* < 0.05; **, *P* < 0.01; ***, *P* < 0.001

**Fig. 2:**
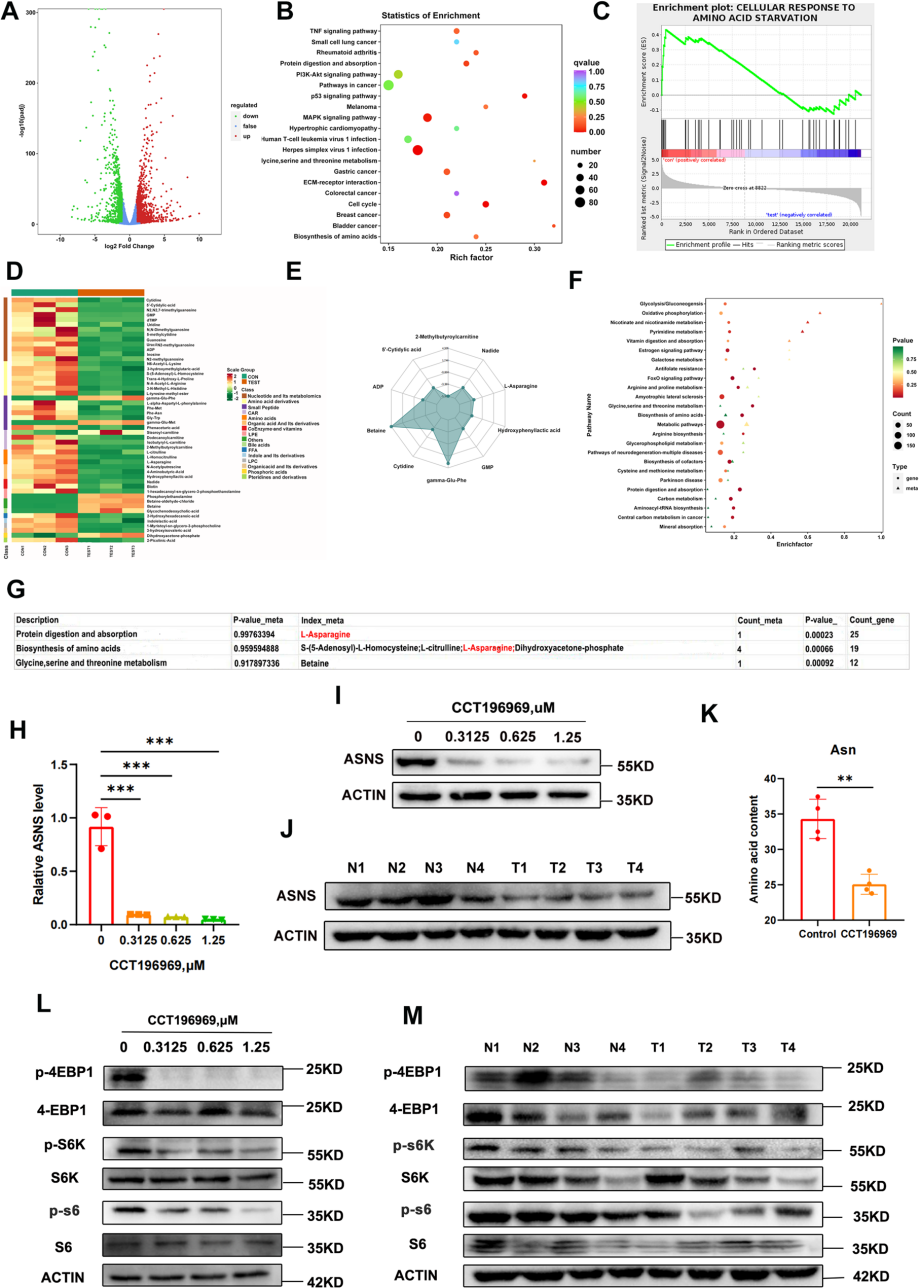
CCT196969 inhibits TNBC by down-regulating ASNS to inhibit asparagine synthesis and its downstream mTORC pathway. **A**. The volcano map showed 2614 differential genes (|log2Fold Change|≥1, FDR < 0.05), with up-regulated genes in red markers, down-regulated genes in green markers, and no significant differential genes in blue markers. **B**. KEGG enrichment analysis showed the 20 pathways with the most significant differences. **C**. GSEA enrichment map of amino acid starvation. **D**. Differential metabolite clustering heat map (Fold_Change ≥ 2, Fold_Change ≤ 0.5 or VIP ≥ 1). **E**. Radar map of the top ten metabolites with the largest FC values. **F**. Bubble map of combined transcriptome and metabolome analysis. **G**. The top three of the co-enrichment pathway sorted by P-value. **H**. qPCR was used to detect the mRNA expression of ASNS in 4T1 cells treated with different concentrations of CCT196969 for 48 h. **I**. Western blot analysis of ASNS protein expression in 4T1 cells treated with different concentrations of CCT196969 for 48 h. **J**. Western blot detection of ASNS protein expression in tumor tissues of mice in Control group (N) and CCT196969 treatment group (T). **K**. The content of asparagine in blood of mice was analyzed by mass spectrometry. **L**. Western blot analysis of mTORC1 pathway protein expression in 4T1 cells treated with different concentrations of CCT196969 for 48 h. **M**. Western blot analysis of mTORC1 pathway protein expression in mouse tumor tissues. The samples used for **J**, **K** and **M** are from Fig. 1. All membranes were cut before antibody incubation, and all the following Western blotting figure legends are the same as this one. Data were mean ± SD, *P < 0.05, **P < 0.01, ***P < 0.001

We treated human normal breast epithelial tissue cells MCF-10 A and three TNBC cells with increasing concentrations of CCT196969 for 48 h, and CCK-8 assay showed significant inhibition on 4T1, MDA-MB-231 and HCC-1806 cells, with IC50 of 0.85 µM, 0.28 µM and 1.9 µM, respectively, while the IC50 of MCF-10 A (3.4 µM) was higher than three of them (Fig. 1A, Supplementary Fig. 1A). Cell phenotype experiments showed that TNBC cell proliferation (Fig. 1B-C, Supplementary Fig. 1B-C), invasion, and migration (Fig. 1D-E, Supplementary Fig. 1D-E) were all significantly inhibited by CCT196969 and apoptosis was moderately induced (Fig. 1F-G, Supplementary Fig. 1F-G). Subsequently, we conducted in vivo experiments and constructed a mouse in situ breast tumor model to observe the growth of mouse tumors. Through measurement and analysis, the tumor volume and weight of mice in the CCT196969 treatment group were significantly lower than those in the Control group (Fig. 1H-J). However, there was no significant difference in body weight between the two groups of mice (Fig. 1K). Moreover, we established a mouse model of TNBC lung metastasis in Balb/c mice by tail vein injection with 4T1-luc cells. After treating the mice with CCT196969 for 11 days, in vivo imaging of the mice was performed using the IVIS Spectrum system (Supplementary Fig. 2A). Meanwhile, the lung tissues of mice were taken by lung perfusion method, fixed and stained with HE, and the tumor nodules were counted under a microscope. The results showed that lung fluorescence value and the number/area of pulmonary metastatic nodules in the CCT196969 treatment group was significantly less than that in the control group (Supplementary Fig. 2B-D). Additionally, there was no significant difference in body weight between the two groups of mice (Fig. 2E). These results suggest that CCT196969 can safely and significantly inhibit the proliferation and metastasis of TNBC cells both in vivo and in vitro, and is a potential TNBC inhibitor.

### CCT196969 inhibits TNBC by down-regulating ASNS to limit asparagine synthesis and then inhibit mTOR signaling pathway

To explore the mechanism of CCT196969 inhibiting TNBC, transcriptomic analysis was performed on 4T1 cells before and after CCT196969 treatment, and 2614 differential genes (1124 up-regulated and 1490 down-regulated) were screened (Fig. 2A). Then, KEGG analysis was performed on the differential genes, and the results showed that CCT196969 was closely related to Protein digestion and absorption as well as Biosynthesis of amino acids (Fig. 2B), and GSEA analysis showed that CCT196969 was positively related to amino acid starvation (Fig. 2C). We then used targeted metabolomics and detected more than 160 metabolites. Amino acid-related metabolites were significantly down-regulated. Moreover, among the top ten changed metabolites, asparagine was the only amino acid and down-regulated (Fig. 2D-E). Combined transcriptome and metabolome analysis showed asparagine and ASNS were involved in the most significant enrichment of pathways—Protein digestion and absorption as well as Biosynthesis of amino acids (Fig. 2F-G). Therefore, we suspected that CCT196969 might inhibit TNBC by restricting asparagine metabolism. ASNS is an enzyme that converts aspartic acid to asparagine, and its expression is positively correlated with the content of asparagine, which may play an important role in the inhibition of TNBC by CCT196969. qPCR and Western blot confirmed that CCT196969 inhibited ASNS expression at both transcriptional and protein levels (Fig. 2H-J) and reduced blood asparagine levels in mice (Fig. 2K). Studies have shown that the mTOR pathway promotes tumor proliferation and metabolism [13], and that asparagine can activate mTORC1 through Arf1 [6]. Western blot analysis showed that CCT196969 inhibited phosphorylation of mTORC1 pathway (p-4EBP1, p-S6K, p-S6) in 4T1 cells and tumor tissues (Fig. 2L-M). In addition, we conducted IHC experiments on the expression of ASNS, mTORC1 pathways and Ki67 in tumor tissues (the tumor tissues were from Fig. 1H) treated with/without CCT196969. The results showed that the expressions of ASNS, mTORC1 pathways (p-S6K, p-S6 and p-4EBP1) and Ki67 in the tumor tissues treated with CCT196969 were significantly lower than that in the control group (Supplementary Fig. 3A). These results all showed that CCT196969 inhibited the synthesis of asparagine and its downstream mTOR signaling pathway by down-regulating ASNS, thus inhibiting TNBC.

### TNBC is more effectively suppressed by CCT196969 combined with ASNase

To verify whether decreased asparagine synthesis mediated the inhibitory effect of CCT196969 on TNBC, 4T1 cells treated with CCT196969 were cultured in normal medium and high concentration asparagine medium, respectively. The results showed that CCT196969 significantly inhibited cell proliferation, invasion, and migration, but the addition of asparagine reversed this inhibition (Supplementary Fig. 4A-C), suggesting that the reduction of asparagine played a key role in the suppression of TNBC by CCT196969. Currently, ASNase is effective in reducing the level of circulating asparagine and has been approved for the treatment of acute lymphoblastic leukemia (ALL) [14]. In vivo experiments showed that the tumor volume and weight of mice treated with CCT196969 combined with ASNase were smaller than those treated alone (Supplementary Fig. 4D-G), and there was no significant difference in body weight between the groups (Supplementary Fig. 4H). The results showed that CCT196969 combined with ASNase could inhibit triple-negative breast cancer more effectively without obvious side effects.

### CCT1969696 mediates the down-regulation of ASNS by increasing RXRA levels

**Fig. 3:**
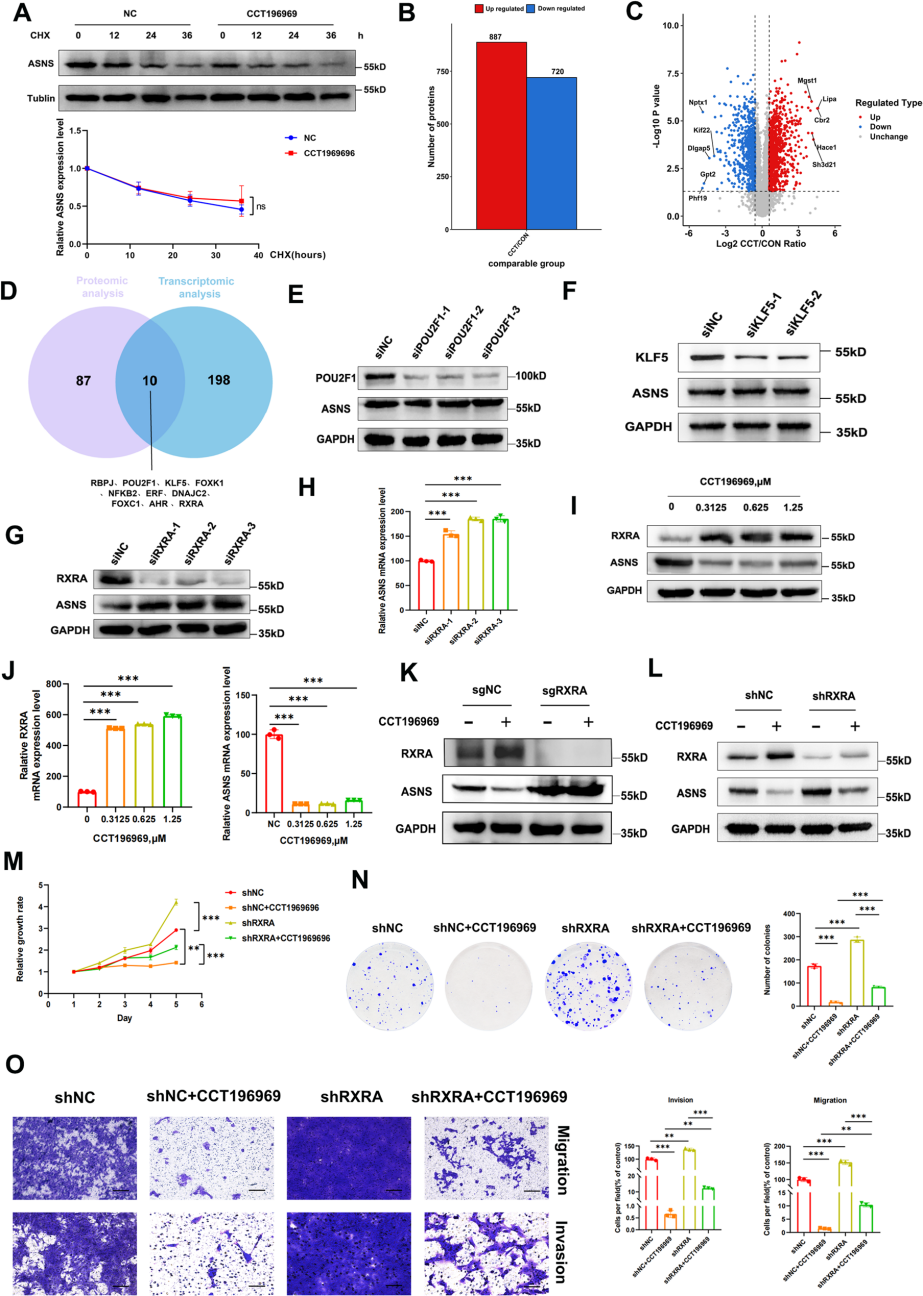
CCT196969 inhibits ASNS expression by up-regulating RXRA. **A**. 4T1 cells treated with CCT196969 for 48 h were then treated with CHX, and the protein was collected after 0, 12, 24, 36 h, respectively. Western blot analysis was performed to detect the degradation of ASNS protein (3 repetitions, the figure above is representative and the figure below is statistical). **B**. Statistical maps of proteomic analysis between control group and CCT196969 treatment group. **C**. Differential protein volcano map (red: significantly up-regulated, blue: significantly down-regulated, gray: no significant difference). **D**. Venn diagram of transcription factors for combined analysis of proteome and transcriptome. **E**. Western blot analysis of ASNS protein expression after POU2F1 knockdown. **F**. Western blot analysis of ASNS protein expression after KLF5 knockdown. **G**. Western blot analysis of ASNS protein expression after RXRA knockdown. **H**. qPCR was used to detect ASNS mRNA expression after RXRA knockdown. **I**. Western blot analysis of RXRA and ASNS protein expression after CCT196969 treatment. **J**. mRNA expression of RXRA and ASNS after CCT196969 treatment was detected by qPCR. **K**-**L**. Western blot analysis of ASNS protein expression after CCT196969 treatment in sgNC-4T1 and sgRXRA-4T1 cells (**K**), shNC-4T1 and shRXRA-4T1 cells (**L**). **M**-**O**. shNC-4T1 and shRXRA-4T1 cells were treated or not treated with CCT196969, and the following experiments were performed: **M**. CCK-8 experiment (3 independent experiments). **N**. clone formation experiment (3 repetitions, representative graph on the left, statistical graph on the right). **O**. Transwell experiment (3 independent experiments, representative graph on the left and statistical graph on the right). Scale bars, 20 μm. Data were mean ± SD, *P < 0.05, **P < 0.01, ***P < 0.001

CCT196969 could reduce the transcription and protein levels of ASNS, to further clarify how CCT196969 inhibited ASNS, we first treated 4T1 cells with CHX, and found that the half-life of ASNS was not changed by CCT196969 (Fig. 3A), indicating that CCT196969 did not regulate post-translational levels of ASNS. Therefore, we focused on the regulation of transcription factors by CCT196969. Proteomic analysis showed that 887 proteins were up-regulated and 720 proteins were down-regulated after treatment with CCT196969 (Fig. 3B-C). Combined with the transcriptome data, we found that there were 10 transcription factors that changed both at the transcriptional level and protein level after CCT196969 treatment, and the changes were in the same direction—RBPJ, POU2F1, KLF5, FOXK1, NFKB2, ERF, DNAJC2, FOXC1, AHR, RXRA (Fig. 3D). According to the hTF transcription factor analysis website, only POU2F1, KLF5 and RXRA can bind to ASNS promoter. POU2F1 is a transcriptional activator that is highly expressed in a variety of cancers and promotes tumor development [15–18]. KLF5 is a transcriptional activator that is often used as a therapeutic target and prognostic marker for cancer [19]. However, RXRA usually acts as a transcriptional repressor and is involved in processes such as gene expression, signal transduction and cell senescence [20, 21]. Further experiments showed that knockdown of POU2F1 and KLF5 did not affect ASNS expression (Fig. 3E-F), while knockdown of RXRA significantly increased the mRNA and protein levels of ASNS (Fig. 3G-H). After treatment with CCT196969, the expression of RXRA was up-regulated and the expression of ASNS was down-regulated in both protein and mRNA levels (Fig. 3I-J), indicating that CCT196969 might inhibit ASNS by up-regulating RXRA. To further verify this mechanism, we constructed a stable 4T1 cell line with RXRA knockout/knockdown, and found that ASNS expression was down-regulated after CCT196969 treatment, and combined RXRA knockout/knockdown could reverse the down-regulation of ASNS expression, the distinction was that CCT196969 had no effect on the expression of ASNS in the 4T1 cells knocked out by RXRA (Fig. 3K-L). Clone formation, CCK-8 and transwell experiments showed that compared with CCT196969 alone, combined RXRA knockdown significantly weakened the inhibitory effect on tumor cell proliferation, invasion and migration (Fig. 3M-O), which further confirmed that CCT196969 inhibited TNBC by up-regulating RXRA and down-regulating ASNS.

### The − 1114/-1104 region of the ASNS promoter is a key region in the regulation of ASNS transcription by RXRA

According to the analysis of TCGA database, the expression of RXRA in breast cancer tissues was lower than that in normal tissues, and the expression was lowest in Basal like subtype (mostly TNBC) (Supplementary Fig. 5A), suggesting that RXRA might play a role in cancer suppression in TNBC. In contrast, ASNS expression was significantly up-regulated in breast cancer, with the highest expression in Basal-like subtypes (Supplementary Fig. 5B). TCGA data further confirmed that RXRA was negatively correlated with ASNS (Supplementary Fig. 5C). In order to explore the binding sites of RXRA and ASNS promoter, we obtained the mouse ASNS promoter sequence from NCBI, and predicted the top 5 sites of RXRA and ASNS promoter binding through JASPAR website. Then mutation design was performed (Supplementary Fig. 5D) and five mutant plasmids were constructed (Supplementary Fig. 5E). Dual luciferase assay showed that ASNS promoter activity in shRXRA-4T1 cells was significantly higher than that in shNC-4T1 cells (Supplementary Fig. 5F). When the wild type and mutant promoter plasmids of ASNS were transferred into 4T1 cells, it was found that the activity of ASNS promoter was significantly reduced after mutation in the − 1114/-1104 region (mut4), and there was no further change in activity after CCT196969 treatment (Supplementary Fig. 5G). CHIP-qPCR confirmed that RXRA was bound to the ASNS promoter − 1114/-1104 region, and the binding increased after CCT196969 treatment (Supplementary Fig. 5H).

### CCT196969 up-regulates RXRA by targeting HDAC5 inhibition, thereby reducing the expression of ASNS

**Fig. 4:**
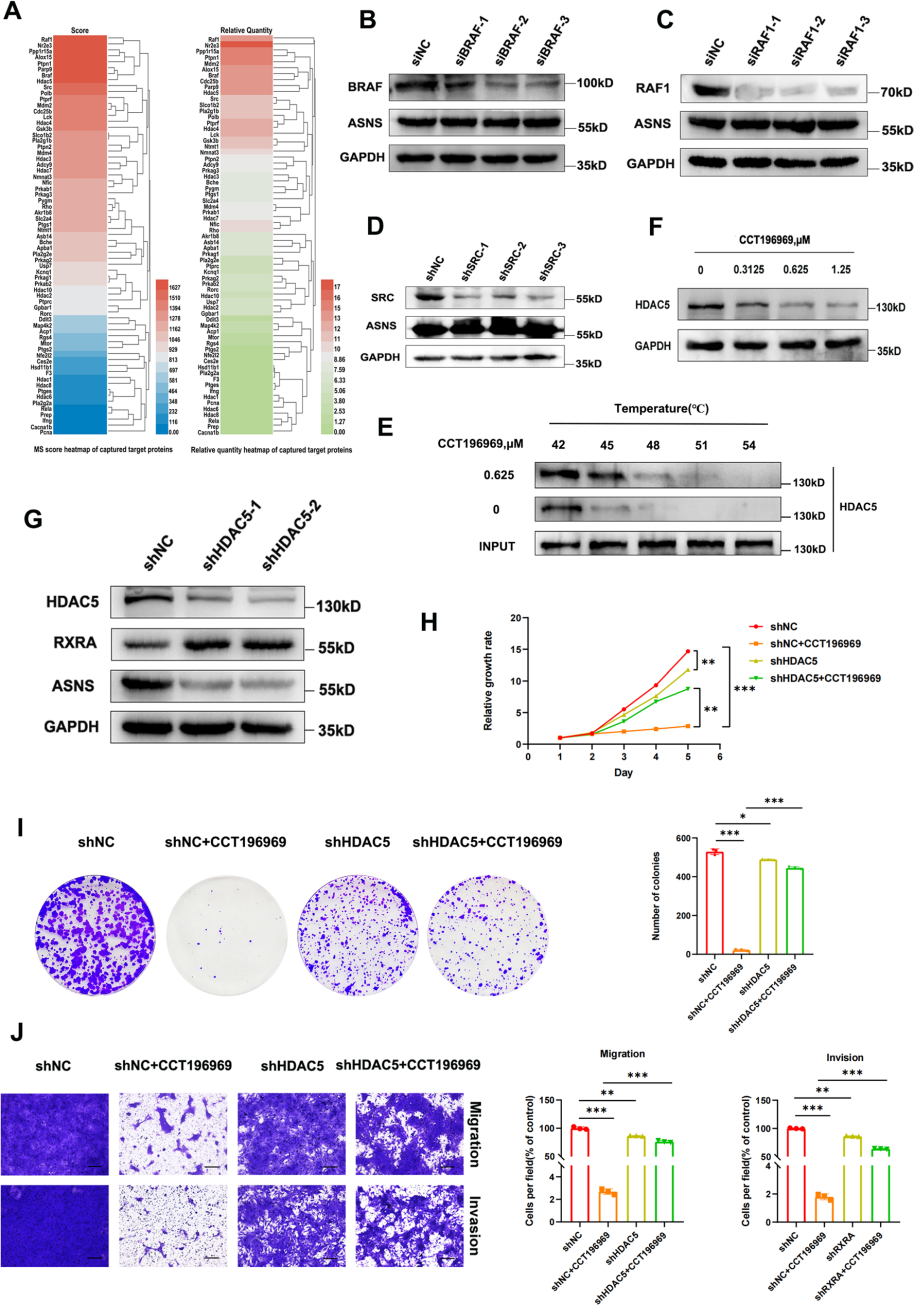
CCT196969 up-regulates RXRA by targetly inhibiting HDAC5, and then down-regulates ASNS expression, inhibiting TNBC. **A**. CCT196969 was fixed with 3D optical crosslinking chip, 4T1 cell lysate was circulated, targeted proteins were identified by LC-MS, and heat maps were generated by clustering according to Score and Relative Quantity. Western blot was used to detect the ASNS protein expression after BRAF knockdown (**B**)/RAF1 knockdown (**C**)/SRC knockdown (**D**), the thermostability of HDAC5 (**E**)/HDAC5 protein expression (**F**) before and after CCT196969 treatment and the protein expression of RXRA and ASNS after HDAC5 knockdown (**G**). **H**-**J**. shNC-4T1 and shHDAC5-4T1 cells were treated or not treated with CCT196969, and the following experiments were performed: **H**. CCK-8 experiment (3 independent experiments). **I**. Clone formation experiment (3 repetitions, representative graph on the left, statistical graph on the right). **J**. Transwell experiment (3 independent experiments, representative graph on the left, statistical graph on the right). Scale bars, 20 μm. Data were mean ± SD, *P < 0.05, **P < 0.01, ***P < 0.001

Through target capture experiments and mass spectrometry analysis of CCT196969, we identified its directly interacting protein (Fig. 4A), excluding RXRA, indicating that CCT196969 indirectly regulates RXRA through other targets. CCT196969 is an oral pan-RAF inhibitor that also targets SRC family kinases [12]. The top ten directly interacting proteins (Score greater than 1500) included RAF1, BRAF, and SRC, but ASNS expression did not change after they were knocked down (Fig. 4B-D). Among the other top 10 target proteins, only HDAC5 has transcriptional regulatory activity. HDAC5 belongs to the Class II histone deacetylase family, which inhibits transcription by deacetylating lysine residues of core histones (H2A, H2B, H3, and H4) and non-histone proteins, and is a potential target for anticancer drugs [22]. The surface plasmon resonance (SPR) experiment revealed a robust interaction between CCT196969 and HDAC5 protein, exhibiting an affinity constant of 32.10 nM (Supplementary Fig. 6A). Additionally, CETSA showed that the thermal stability of HDAC5 was enhanced after CCT196969 treatment (Fig. 4E, Supplementary Fig. 6B-C), and the level of HDAC5 protein was inhibited by CCT196969 (Fig. 4F). Western blot results showed that knocking down of HDAC5 up-regulated the expression of RXRA protein and decreased the expression of ASNS protein (Fig. 4G, Supplementary Fig. 6D-E). After HDAC5 knockdown alone, cell proliferation, invasion and migration abilities were weakened. More importantly, HDAC5 knockdown combined with CCT196969 treatment group significantly reduced the inhibitory effect on TNBC compared with CCT196969 treatment group alone (Fig. 4H-J). Therefore, it is reasonable to speculate that HDAC5 is the direct target of CCT196969 in TNBC cells, and CCT196969 up-regulates RXRA by targetly inhibiting HDAC5, thereby reducing the expression of ASNS.

### HDAC5 decreases the transcription level of RXRA and reduces the protein stability of RXRA through the ubiquitin-proteasome pathway

**Fig. 5:**
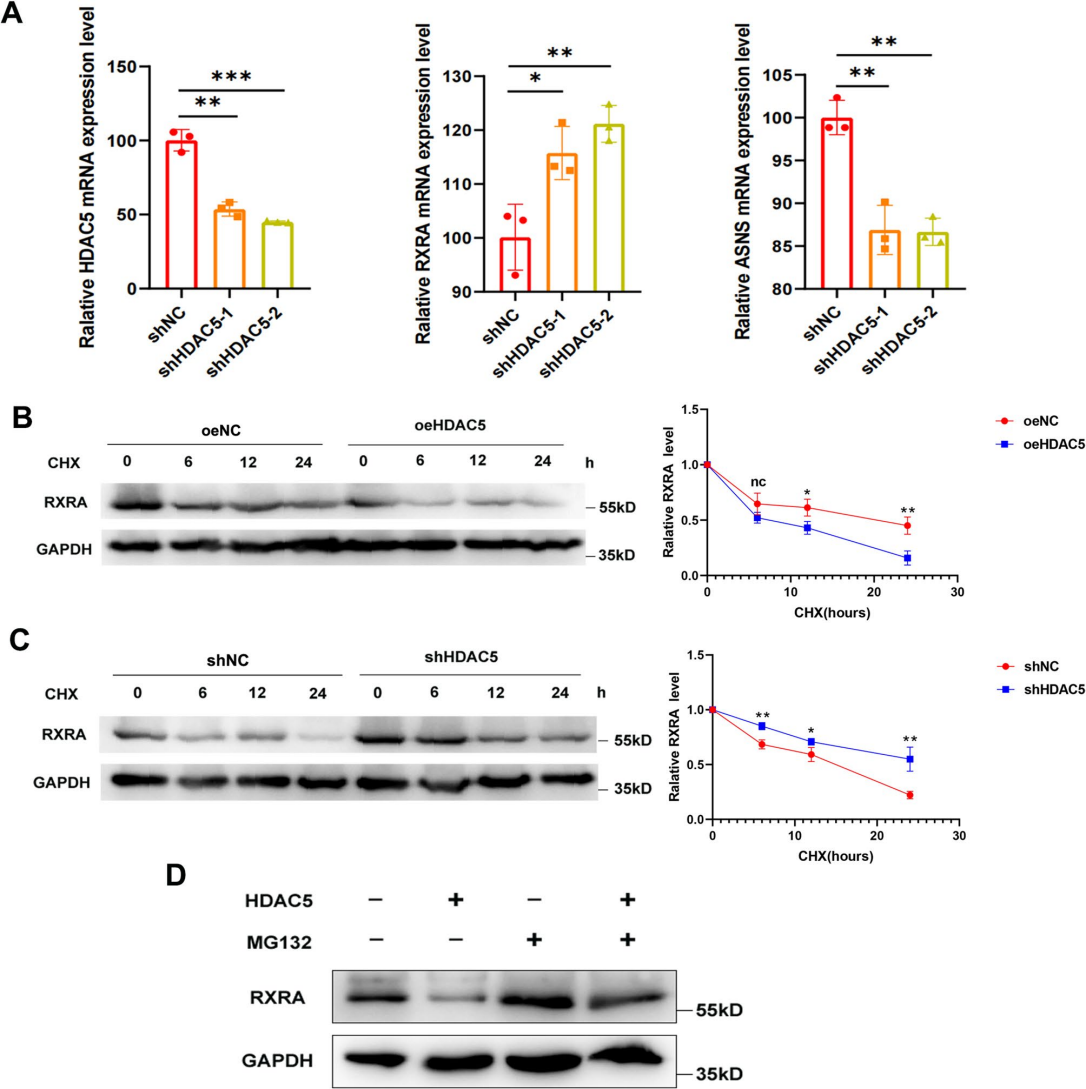
HDAC5 down-regulates RXRA expression at the transcriptional level and reduces RXRA protein stability through the ubiquitin-proteasome pathway. **A**. The mRNA expression of RXRA and ASNS after HDAC5 knockdown was detected by qPCR. **B**. After overexpression of HDAC5, CHX was added to detect the change of RXRA protein level by Western blot (3 repetitions, representative figure on the left and statistical figure on the right). **C**. shNC-4T1 and shHDAC5-4T1 cells were treated with CHX, and the changes of RXRA protein levels were detected by Western blot (3 repetitions, representative figure on the left and statistical figure on the right). **D**. The 4T1 cells overexpressing HDAC5 were treated with MG132, and the changes of RXRA protein levels were detected by Western blot. Data were mean ± SD, *P < 0.05, **P < 0.01, ***P < 0.001

Through previous studies, we confirmed HDAC5 as the key target of CCT196969 in inhibiting TNBC. Next, we began to explore the regulatory relationship between HDAC5 and RXRA. When HDAC5 was knocked down, qPCR showed that RXRA mRNA expression increased and ASNS mRNA expression decreased (Fig. 5A), indicating that HDAC5 inhibited RXRA transcription and thus up-regulated ASNS mRNA level. Western blot has confirmed that knocking down HDAC5 up-regulated RXRA protein expression (Fig. 4G and Supplementary Fig. 6D-E). Further experiments showed that overexpression of HDAC5 accelerated the degradation of RXRA protein (Fig. 5B), while knocking down of HDAC5 delayed the degradation of RXRA protein (Fig. 5C). Subsequently, 4T1 cells overexpressing HDAC5 were treated with MG132. MG132 is a protease inhibitor that inhibits intracellular protease activity and enhances protein stability in cells. Western blot showed that the overexpression of HDAC5 inhibited the expression of RXRA protein level, but the inhibition was reduced after MG132 treatment. Therefore, we can conclude that HDAC5 reduced the stability of RXRA protein through the ubiquitin-proteasome pathway (Fig. 5D).

### Interaction between HDAC5 and RXRA

**Fig. 6:**
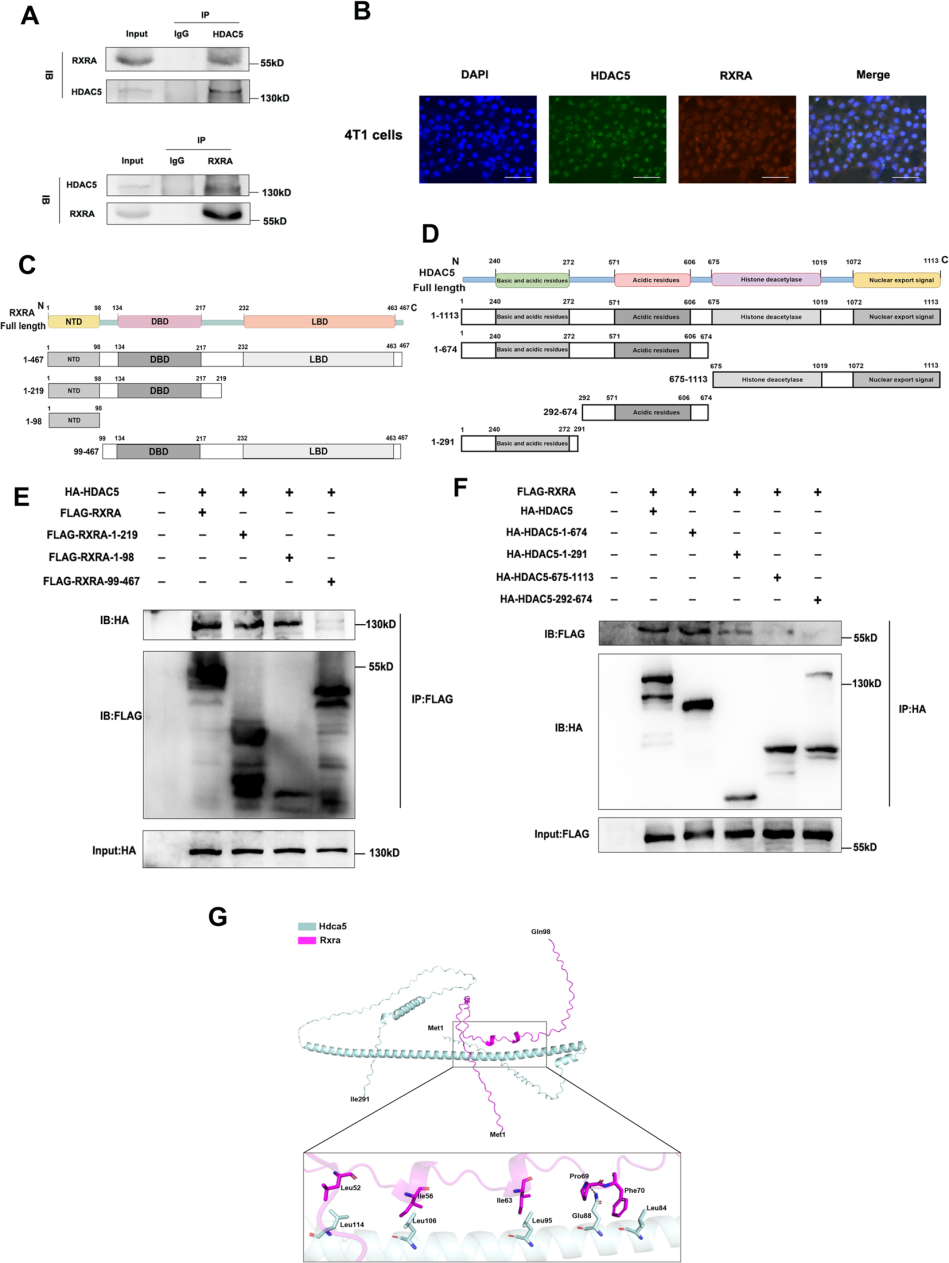
Interaction between HDAC5 and RXRA. **A**. CO-IP experiment of HDAC5 and RXRA in 4T1 cells. **B**. Immunofluorescence co-localization of HDAC5 and RXRA in 4T1 cells. Scale bars, 75 μm. **C**-**D**. Structure diagram and truncated design of HDAC5 and RXRA. **E**. Full-length HDAC5 and RXRA truncated segments were co-transfected into 293T cells, and the key binding domain of RXRA was detected by CO-IP. **F**. Full-length RXRA and HDAC5 truncated segments were co-transfected into 293T cells, and the key binding domain of HDAC5 was detected by CO-IP. **G**. Simulation of HDAC5 and RXRA binding model by molecular docking

Through endogenous CO-IP experiments, we found that HDAC5 interacts with RXRA (Fig. 6A). Immunofluorescence co-localization also showed that HDAC5 and RXRA were co-localized in the TNBC nucleus (Fig. 6B). To identify the binding region, we constructed a series of amino acid truncated proteins of HDAC5 and RXRA (Fig. 6C-D). CO-IP experiments showed that 1–98 region and 1-219 region of RXRA bind to HDAC5, while regions 99–467 do not bind (Fig. 6E). Similarly, 1-291 region and 1-674 region of HDAC5 bind to RXRA, while 675–1113 region and 292–674 region do not bind (Fig. 6F). Therefore, we preliminarily concluded that HDAC5 interacted directly with RXRA’s 1–98 region through its 1-291 region. Subsequently, we used AlphaFold3 to predict the interaction sites between the 1-291 region of HDAC5 and the 1–98 region of RXRA and visualized them with PYMOL (Fig. 6G). The results showed that they were bound mainly by hydrophobic forces (Leu52-Leu114, Ile56-Leu106, Ile63-Leu95, Phe70-Leu84) and hydrogen bonds (Pro69-Glu88). This further confirmed that HDAC5 interacted directly with RXRA’s 1–98 region through its 1-291 region.

### HDAC5 reduces the acetylation level of RXRA, thereby increasing the ubiquitination level of RXRA and the expression of downstream ASNS

**Fig. 7:**
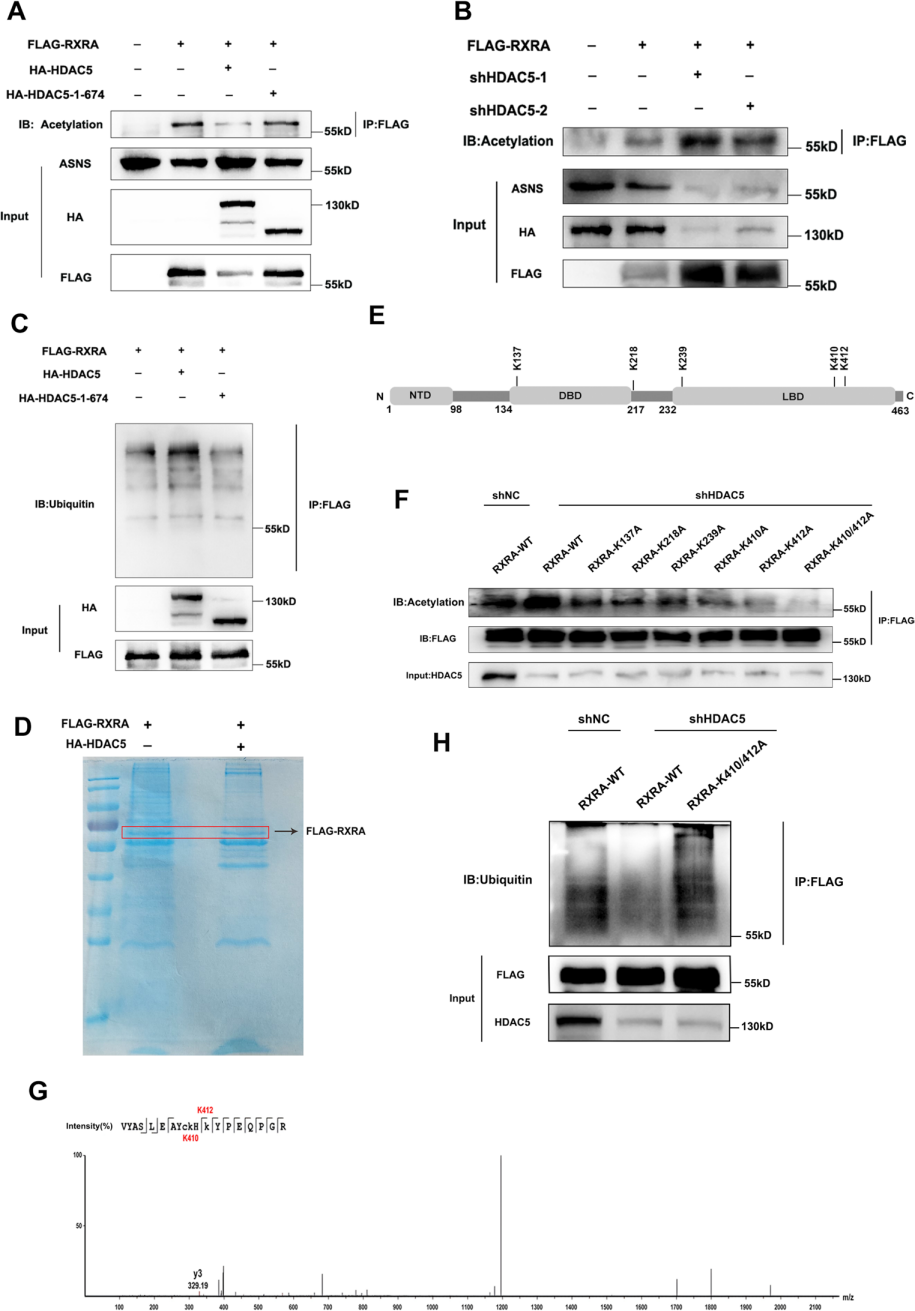
HDAC5 reduces the acetylation level of RXRA, thereby increasing the ubiquitination level of RXRA and the expression of downstream genes ASNS. **A**. Truncated HDAC5 (1-674) or full-length HDAC5 and RXRA were transfected into 293T cells, and the acetylation level of RXRA and the expression level of ASNS were detected by IP assay. **B**. After HDAC5 was knocked down, RXRA acetylation and ASNS expression were detected by IP assay. **C**. truncated HDAC5 (1-674) or full-length HDAC5 and RXRA were transferred into 293T cells, and RXRA ubiquitination and ASNS expression were detected by IP. **D**. HDAC5-HA and RXRA-Flag plasmid were co-transfected into 293T cells, only RXRA-Flag was transfected in the control group, and the protein was extracted 48 h later, and RXRA was enriched with the anti-flag antibody. **E**. Schematic diagram of the lysine site of HDAC5 deacetylated RXRA based on co-immunoprecipitation and mass spectrometry. **F**. RXRA mutant or wild-type plasmids were transfected into 293T cells with HDAC5 knockdown, and RXRA acetylation level was detected by IP. **G**. K410/K412 mass spectrogram of RXRA in the control group. **H**. The mutant or wild-type plasmids of RXRA were transfected into 293T cells with HDAC5 knockdown, and the ubiquitination level of RXRA was detected by IP

As a deacetylase, HDAC5 can inhibit RXRA transcription by deacetylating histones on RXRA promoters, so is it possible that HDAC5 can also deacetylate RXRA (non-histone proteins). We obtained the deacetylase active region of HDAC5 from the Uniprot database (675–1076 bp), and then truncated this region to construct the HDAC5 truncate segment (1–674 bp), and transfected this truncated or full-length HDAC5 and RXRA into 293T cells. IP experiments showed that HDAC5 reduced the acetylation level of RXRA and up-regulated the expression of ASNS, while when the deacetylase active region was missing, the acetylation level of RXRA increased and the expression of ASNS was reversed (Fig. 7A). On the contrary, knockdown of HDAC5 increased the acetylation level of RXRA and down-regulated the expression of ASNS (Fig. 7B), indicating that HDAC5 affected the expression of ASNS by regulating the acetylation level of RXRA. It has been reported that acetylation modification might affect ubiquitination modification. We found that wild-type HDAC5 promoted RXRA ubiquitination, while the level of RXRA ubiquitination decreased significantly when the deacetylase active region was absent (Fig. 7C), suggesting that the deacetylase active region of HDAC5 was critical for promoting RXRA ubiquitination. In order to find the key lysine site in RXRA that was deacetylated by HDAC5, we co-transfected HDAC5-HA and RXRA-Flag plasmid into 293T cells, while only RXRA-Flag plasmid was transfected in the control group, and co-immunoprecipitation and mass spectrometry were performed 48 h later (Fig. 7D). Through further analysis and screening, we found five lysine sites where RXRA was deacetylated in cells co-transfected with HDAC5-HA and RXRA-Flag plasmids compared to the control group (Fig. 7E). After these lysines were mutated to alanine, it was found that the acetylation level of RXRA was significantly reduced when K410 and K412 were mutated at the same time (Fig. 7F), indicating that K410 and K412 were key deacetylation sites for HDAC5 to regulate RXRA (Fig. 7G). Subsequently, we transfected RXRA WT or K410/K412 mutant into 293T cells with/without knockdown of HDAC5, and the ubiquitination level of RXRA was detected by Western blot. The results showed that when RXRA K410 and K412 mutated, the ubiquitination level of RXRA increased compared with the RXRA WT group in 293T cells with knockdown of HDAC5 (Fig. 7H). These results indicate that HDAC5 reduces the acetylation level of RXRA, thereby increasing the ubiquitination level of RXRA.

### There is a positive correlation between the expression of HDAC5 and ASNS

**Fig. 8:**
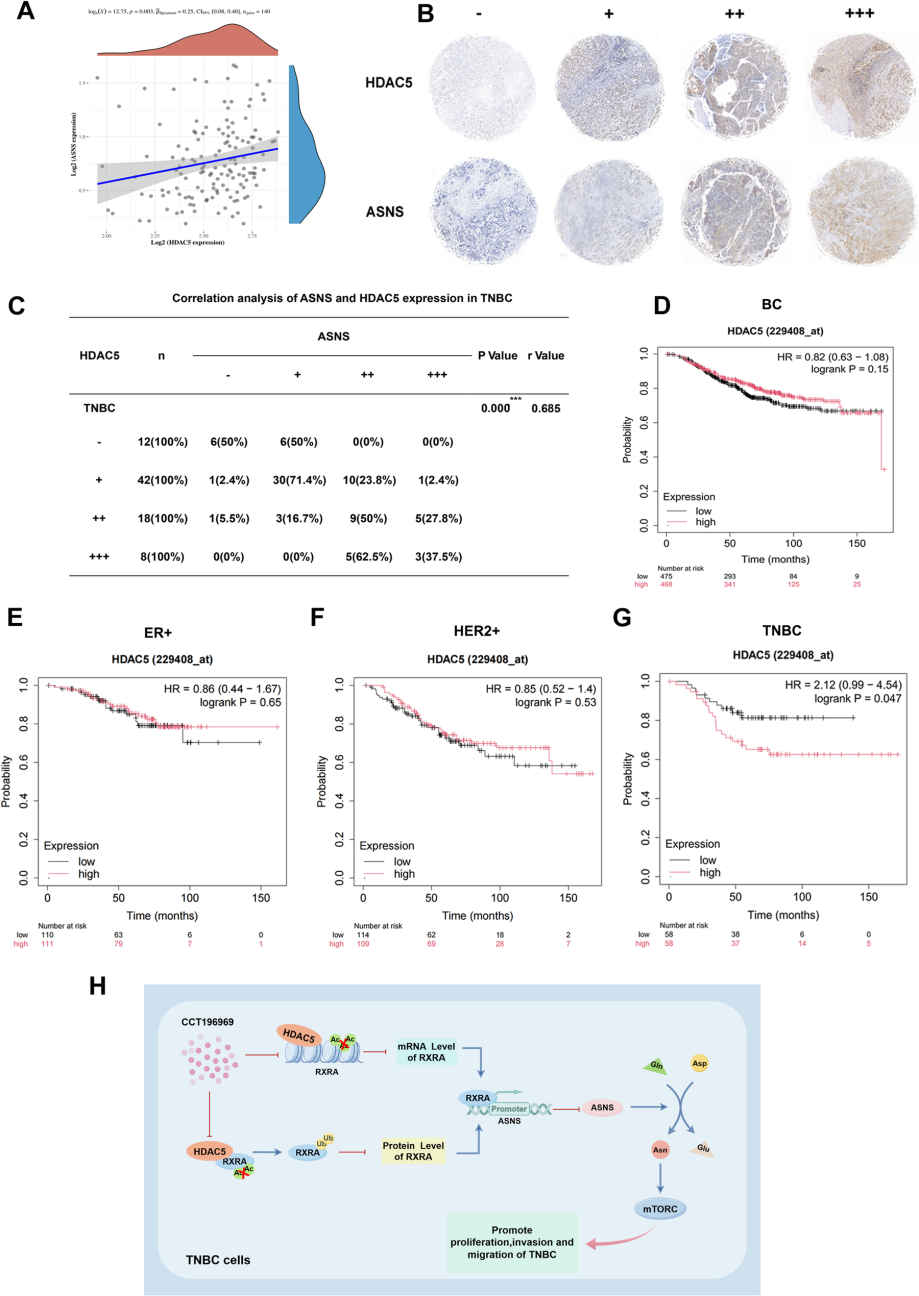
There is a positive correlation between the expression of HDAC5 and ASNS. **A**. The correlation between HDAC5 and ASNS mRNA levels was analyzed by TCGA database. **B**. Immunohistochemical representation of HDAC5 and ASNS in cancer tissue chips of 80 TNBC patients. **C**. Correlation between HDAC5 expression and ASNS expression in 80 TNBC patients. **D**-**G**. TCGA database was used to analyze the relationship between HDAC5 expression level and clinical prognosis (OS) in BC patients (**D**), ER-positive BC patients (**E**), HER2-positive BC patients (**F**) and TNBC patients (**G**). H. Molecular mechanism diagram, CCT196969 down-regulates asparagine synthesis by targeting the HDAC5/RXRA/ASNS axis and further inhibits the mTORC pathway. Ultimately, CCT196969 inhibited the proliferation, invasion and migration of triple-negative breast cancer

According to the analysis of TCGA database, there was a significant positive correlation between HDAC5 and ASNS at mRNA level (Fig. 8A). To verify the correlation of protein levels, immunohistochemical staining of HDAC5 and ASNS was performed on TNBC tissue chips, and it was found that HDAC5 was positively correlated with ASNS expression in cancer tissues of TNBC patients (Fig. 8B-C), which was consistent with the results of TCGA. In addition, TCGA data analysis showed that HDAC5 expression level was not significantly correlated with the prognosis of overall BC and ER-positive or HER2-positive subtypes, while in TNBC patients, patients with low HDAC5 expression had longer OS and better prognosis (Fig. 8D-G), suggesting that HDAC5 may be a potential therapeutic target for TNBC.

## Discussion

TNBC, a subtype of breast cancer characterized by the absence of ER, PR, and HER2 expression, is associated with a significantly poor prognosis. The recurrence rate and mortality of TNBC are higher than those of other breast cancer subtypes, and patients do not benefit from endocrine therapy or HER2-targeted therapy, particularly within the first 3 to 5 years following diagnosis [23]. Consequently, chemotherapy remains the standard treatment regimen for non-surgical management of TNBC [24]. Currently, potential treatment strategies for TNBC include drugs targeting the cell cycle, DNA repair pathways, androgen receptor signaling pathways, and various kinases [25–28]. The small molecule inhibitor CCT196969 has been shown to inhibit tumor cell proliferation, survival, and metastasis in melanoma and colorectal cancer by targeting and inhibiting the RAS/RAF/MEK/ERK signaling pathway. Additionally, it may offer potential advantages in treating brain metastatic tumors due to its ability to penetrate the blood-brain barrier [12]. However, the inhibitory effect of CCT196969 on TNBC and the specific mechanism involved remains unclear. This study provides compelling evidence for the anti-TNBC effects of CCT196969.

Through CCK-8 assay, clone formation assay, transwell assay, and apoptosis assay, we demonstrated that CCT196969 effectively inhibited the proliferation, invasion, and migration of TNBC cells, while moderately inducing cell apoptosis. Notably, its proliferation inhibitory effect surpassed that of many clinical therapeutic agents, including cisplatin and gemcitabine [29]. Furthermore, in vivo experiments showed that CCT196969 significantly inhibited the growth and the lung metastasis of TNBC, suggesting that CCT196969 is a potential inhibitor against TNBC. Notably, by comparing the IC50 results of CCT196969 in three TNBC cells and MCF-10 A, We considered that the potential therapeutic window of the compound in vitro might be within 2 µM (because HCC-1806 cells were less sensitive to CCT196969 than 4T1 cells and MDA-MB-231 cells). In the in vivo study, we found that 10 mg/kg/d CCT196969 (gavage) had little effect on the body weight of the mice, indicating that this dose is safe and effective for the treatment of mice. Subsequent transcriptomic, metabolomic, Western blot and IHC analyses revealed that CCT196969 downregulated asparagine as well as the mRNA and protein expression levels of ASNS, along with significantly reducing the expression of downstream targets of mTORC1 pathway, such as p-4EBP1, p-S6K, and p-S6. ASNS, an enzyme involved in asparagine metabolism, plays a crucial role in the synthesis of asparagine within cells, which is vital for the growth and development of tumor cells [30]. The deletion of ASNS in cells can lead to varying degrees of tumor cell apoptosis, autophagy, and cell cycle arrest [31–33]. ASNase, which targets asparagine, has been successfully utilized in the treatment of leukemia [33]. In our study, we observed that the inhibitory effect of CCT196969 on TNBC cells was effectively reversed when sufficient amounts of asparagine were administered to the cells treated with CCT196969. In addition, the combination of CCT196969 with ASNase demonstrated a superior inhibitory effect on TNBC in mice compared to ASNase alone, without exhibiting significant toxic side effects. Consequently, our study indicated that CCT196969 can inhibit TNBC by down-regulating ASNS, thereby reducing asparagine synthesis and its downstream mTOR signaling pathway.

An important finding of this study is that CCT196969 does not affect the half-life of ASNS, but it inhibits the mRNA level of ASNS by up-regulating the transcription factor RXRA. RXRA is a retinoid receptor that mediates retinoid biological effects by participating in retinoid-mediated gene activation. It can function as either a transcriptional suppressor or activator by binding to specific sequences in gene promoters [34]. Studies have shown that the expression of RXRA increases during normal cell differentiation but is typically suppressed in cancer cells [35]. Compared to normal tissues, the content of RXRA in prostate cancer is relatively low, and this low expression is negatively correlated with relapse-free survival and an increased risk of distant recurrence following radiotherapy. Additionally, it has been confirmed that the knockdown of RXRA can induce radiation resistance [36], and its deletion can promote leukemia growth in mice [37]. RXRA is increasingly recognized as a promising candidate for the prevention and treatment of various human cancers. In fact, it has been utilized in clinical trials, particularly in evaluating treatments for acute promyelocytic leukemia and in the prevention of head and neck, cervical, and lung cancers [38]. However, the role of RXRA in TNBC remains unclear. The TCGA database indicates that the expression of RXRA in TNBC tissues is lower than in normal tissues and is negatively correlated with ASNS expression. In our study, RXRA acts as a transcriptional suppressor and binds to the − 1114/-1104 region of the ASNS promoter to inhibit ASNS expression. Furthermore, when RXRA was knocked down in 4T1 cells, the proliferation, invasion, and migration abilities of these cells are significantly enhanced, and the loss of RXRA markedly reduce the inhibitory effect of CCT196969 on TNBC.

Another important finding of this study is that HDAC5 serves as the key and direct target for CCT196969 in TNBC cells, as demonstrated through target capture experiments, CETSA experiments, SPR experiments and so on. HDAC5, a histone deacetylase, utilizes its deacetylase activity to remove acetyl groups from lysine residues on histone and non-histone, leading to transcriptional inhibition of downstream genes [22]. The dysregulation of histone deacetylases is highly associated with cancer progression. Previous studies have indicated that histone deacetylase inhibitors (HDACi) can reduce tumor formation and induce intrinsic apoptosis in breast cancer cells by targeting HDAC5, which relies on the activation of the intrinsic apoptosis pathway involving caspase 9/3 signaling [39]. Currently, several HDACi have been clinically implemented or are undergoing clinical trials as potential anti-tumor agents. During our investigation into the target of CCT196969, we discovered that CCT196969 does not modulate the expression of ASNS via BRAF, RAF1, or SRC which has been reported as the targets of CCT196969, and CCT196969 does not directly affect RXRA. Instead, for the other genes with high scores -NR2E3, PPP1R15A, ALOX15, PTPN1, PARP9, HDAC5 and POLB, HDAC5 is the only factor with a transcriptional regulatory effect. And we also found that CCT196969 directly targets HDAC5 through CETSA. CCT196969 upregulates RXRA by directly targeting HDAC5, subsequently leading to a decrease of ASNS expression. Furthermore, the knockdown of HDAC5 significantly diminished the effects of CCT196969 on cell proliferation, invasion, and migration. Both the TCGA database and immunohistochemical analyses revealed a positive correlation between HDAC5 and ASNS. Notably, HDAC5 exhibited a significant negative correlation with prognosis in TNBC patients, suggesting that it may represent a promising therapeutic target for TNBC in the future.

However, the regulatory mechanism of HDAC5 on RXRA is complex. Acetylation of histones neutralizes the positive charge of lysine residues, which affects the protein-protein interactions between histones and the negatively charged DNA backbone, resulting in a more relaxed chromatin structure that promotes gene transcription. Conversely, histone deacetylation enhances the interaction between the positively charged lysine residues and DNA, leading to a more condensed chromatin state that inhibits the transcriptional machinery. In our study, we found that HDAC5 might deacetylate the histones of RXRA, inhibiting the transcriptional level of RXRA, potentially depending on the co-regulation of certain factors. In the future, we will further investigate which factors can recruit HDAC5 to bind to the histones of RXRA through CO-IP and Reverse-CHIP experiments. At the post-translational level, HDAC5 reduced the protein stability of RXRA via the ubiquitin-proteasome pathway. Subsequent intracellular immunofluorescence and endogenous CO-IP experiments confirmed that HDAC5 and RXRA interact and co-localize in the nucleus of TNBC cells. Additionally, we constructed a series of amino acid truncated segments based on the structural characteristics of HDAC5 and RXRA. CO-IP experiments and AlphaFold3 software predicted that the interaction between HDAC5’s 1-291 region and RXRA’s 1–98 region primarily occurs through hydrophobic forces. For post-translational level regulation, HDAC5 deacetylated RXRA (non-histone proteins) through its deacetylase active region and increases the expression of downstream genes ASNS. Studies have demonstrated that acetylation and ubiquitination often compete for the same lysine residues on proteins, and modifications through acetylation may influence the addition, removal, or function of ubiquitination [40]. We found that HDAC5 can enhance the ubiquitination level of RXRA, which also depends on its deacetylase active region. Utilizing mass spectrometry and co-immunoprecipitation experiments, we identified the key lysine sites for deacetylation of RXRA are K410 and K412. Overall, HDAC5 can inhibit RXRA through transcriptional repression and post-translational modification, which further clarifies the specific mechanism of CCT196969 inhibiting TNBC. Furthermore, HDAC5 can regulate the transcriptional activity of proteins by directly modulating their deacetylation [41]. For instance, in lung adenocarcinoma, HDAC5-mediated deacetylation of SATB1 can also affect its transcriptional regulatory activity on downstream genes [41]. In pancreatic cancer, HDAC5 inhibits the binding of GATA1 to the promoter region of the downstream gene PLA2G4A by deacetylating the transcription factor GATA1 [42]. Additionally, it has been reported that acetylation of RXRA by p300, an acetyltransferase, can enhance its DNA binding and subsequently increase its transcriptional activity [43]. Therefore, we conclude that HDAC5 may also inhibit the transcriptional activity of RXRA by deacetylation of RXRA protein, however, further research is required to confirm this.

## Conclusions

In summary, HDAC5, functioning as a deacetylase, inhibits the transcription of RXRA. Moreover, at the post-translational level, HDAC5 interacts directly with RXRA’s 1–98 region through its 1-291 region to deacetylate lysine residues at K410 and K412 sites of RXRA protein, depending on its deacetylase activity, which increases the ubiquitin levels of RXRA, thereby reducing the protein stability of RXRA through the ubiquitin-proteasome pathway and subsequently up-regulating the expression of ASNS. However, the small molecule compound CCT196969 inhibits the expression of HDAC5 by directly targeting it, which leads to an increase in the expression level of RXRA. The up-regulated RXRA functions as a transcriptional suppressor, further inhibiting the mRNA level of ASNS by binding to the − 1114/-1104 region of the ASNS promoter, reducing asparagine synthesis, and ultimately inhibiting the proliferation, invasion, and migration of TNBC (Fig. 8H). Our study suggests that CCT196969 can inhibit TNBC, explore new pharmacodynamic functions of the compound, and identify a previously unrecognized mechanism related to asparagine metabolism—the HDAC5/RXRA/ASNS axis, which provides potential candidate targets for TNBC treatment. Furthermore, it offers a theoretical basis for the clinical application of CCT196969 in treating TNBC patients.

## Supplementary Information

Below is the link to the electronic supplementary material.

Supplementary Material 1

## Data Availability

No datasets were generated or analysed during the current study.

## Abbreviations

ASNS: Asparagine synthetase
CETSA: Cell thermal shift assay
CHIP: Chromatin Immunoprecipitation
CO-IP: Co-Immunoprecipitation
EMT: Epithelial-mesenchymal transition
ER: Estrogen receptor
HDAC5: Histone Deacetylase 5
HDACi: Histone deacetylase inhibitors
HER2: Human Epidermal GrowthFactor Receptor 2
IF: Immunofuorescence
PR: Progesterone receptor
RXRA: Retinoid X Receptor Alpha
SFK: SRC-family kinases
SPR: Surface plasmon resonance
TNBC: Triple negative breast cancer
