# Supplementary material for: CCT196969 inhibits TNBC by targeting the HDAC5/RXRA/ASNS axis to down-regulate asparagine synthesis
Source: J Exp Clin Cancer Res. 2025 Aug 8;44:231. doi: 10.1186/s13046-025-03494-5 (PMC12333201; doi:10.1186/s13046-025-03494-5)
Supplement: Supplementary file 1 — Supplementary Material 1 [file 13046_2025_3494_MOESM1_ESM.docx]

Supplementary Table 1，siRNA and shRNA

| Genes | Sequence |
| --- | --- |
| siRXRA-1 | sense: GCUCCUCAGGCAAACACUAUGTT  antisense: CAUAGUGUUUGCCUGAGGAGCTT |
| siRXRA-2 | sense: GCUCCAUAGCUGUGAAAGAUGTT  antisense: CAUCUUUCACAGCUAUGGAGCTT |
| siRXRA-3 | sense: GGUGUAUGCGUCACUAGAAGCTT  antisense: GCUUCUAGUGACGCAUACACCTT |
| shRXRA | GCTCCTCAGGCAAACACTATG (5’ to 3’) |
| siPOU2F1-1 | sense: CCUGCAACCAGCACAGUUUAUCA  antisense: GAUGAUAAACUGUGCUGGUUGCAGGUU |
| siPOU2F1-2 | sense: ACAGUCUAAAUCCAGUGAATT  antisense: UUCACUGGAUUUAGACUGUAC |
| siPOU2F1-3 | sense: AUCUAGCCCAAGUGCUUUGTT  antisense: CAAAGCACUUGGGCUAGAUGC |
| siKLF5-1 | sense: AAGCUCACCUGAGGACUCATT  antisense: UGAGUCCUCAGGUGAGCUUTT |
| siKLF5-2 | sense: GCUCACCUGAGGACUCAUATT  antisense: UAUGAGUCCUCAGGUGAGCTT |
| shSRC-1 | GCGGCTGCAGATTGTCAATAA (5’ to 3’) |
| shSRC-2 | GACAATGCCAAGGGCCTAAAT (5’to 3’) |
| shSRC-3 | GGCCCAAGTCATGAAGAAACT (5’ to 3’) |
| siBRAF-1 | sense: GCUAGAUGCCCUUCAGCAAAGTT  antisense: CUUUGCUGAAGGGCAUCUAGCTT |
| siBRAF-2 | sense: GGAGUUACAUGUUGAAGUACUTT  antisense: AGUACUUCAACAUGUAACUCCTT |
| siBRAF-3 | sense: GCGUUGUAGUACAGAGGUUCCTT  antisense: GGAACCUCUGUACUACAACGCTT |
| siRAF1-1 | sense: GUCAGAAGUUCCUGCUAAAUGTTJ  antisense: CAUUUAGCAGGAACUUCUGACTT |
| siRAF1-2 | sense: GGCAGAGAGACUCGAGUUAUUTT  antisense: AAUAACUCGAGUCUCUCUGCCTT |
| siRAF1-3 | sense: GCAUGGAGAUGUUGCAGUAAATT  antisense: UUUACUGCAACAUCUCCAUGCTT |
| shHDAC5-1 | GGGAACCATCCTTGGAAATCC (5’ to 3’) |
| shHDAC5-2 | GGAGAGCTCAAGAATGGATTT (5’ to 3’) |
| siHDAC5-1 | sense: CGCUAGAGAAAGUCAUCGA  antisense: UCGAUGACUUUCUCUAGCG |
| siHDAC5-2 | sense: GCAUGCGGACGGUAGGCAA  antisense: UUGCCUACCGUCCGCAUGC |

Supplementary Table 2，Sequence for qPCR

| Genes | Sequence |
| --- | --- |
| ASNS | Forward Primer: CCAAGTTCAGTATCCTCTCCAG  Reverse Primer: CTTCATGATGCTCGCTTCCA |
| HDAC5 | Forward Primer: GACAGCTCCCCAGTTTTGGT  Reverse Primer: TGCAGCACGTTTTGCTCCT |
| RXRA | Forward Primer: ATGGACACCAAACATTTCCTGC  Reverse Primer: CCAGTGGAGAGCCGATTCC |

Supplementary Table 3，Primers for ASNS promoter mutation

| Primers | Sequence |
| --- | --- |
| Mut1F | GCTTTTGTTTTATAAAAACTAGACAAATTCTCGAG |
| Mut1R | CTCGAGAATTTGTCTAGTTTTTATAAAACAAAAGC |
| Mut2F | CTTATAGATATCAGATCAAACACTAGAAGAGGCAGAATC |
| Mut2R | GATTCTGCCTCTTCTAGTGTTTGATCTGATATCTATAAG |
| Mut3F | CCACAAAGAGTTTAAAAACGACAAAGGCTAGCTATC |
| Mut3R | GATAGCTAGCCTTTGTCGTTTTTAAACTCTTTGTGG |
| Mut4F | CTTTTTAAAAATTTGATATTCTCAAAACTAAACAAGTGCAGTAC |
| Mut4R | GTACTGCACTTGTTTAGTTTTGAGAATATCAAATTTTTAAAAAG |
| Mut5F | GATTTGTCCGTATGTTCAAAACTCACTGATCTATTTGAAC |
| Mut5R | GTTCAAATAGATCAGTGAGTTTTGAACATACGGACAAATC |

Supplementary Table 4，Sequencing primer for ASNS promoter mutant

| Primers | Sequence |
| --- | --- |
| RVprimer3（Mut1/Mut2） | CTAGCAAAATAGGCTGTCCC |
| Mut3/Mut4 | GACGTCCTATGCTATTC |
| Mut5 | GCTGTGCAGGTTTGGAC |

Supplementary Table 5，Primers for constructing truncated amino acid fragments of HDAC5 and RXRA

| Primers | Sequence |
| --- | --- |
| pcDNA3.1-F | GGTGGCGGATCCGAGCTCGGTACC |
| pcDNA3.1-FLAG-R | GGCTCTAGCTCTGACTACAAAGACCATGACGGTGAT |
| pcDNA3.1-HA-R | GGCTCTAGCTCTTACCCATACGATGTTCCAGAT |
| RXRA-1-3.1-F | GAGCTCGGATCCGCCACCATGGACACCAAACATTTCC |
| RXRA-467-3.1-R  RXRA-98-3.1-R | GTAGTCAGAGCTAGAGCCGGTGGCTTGATGTGGTGCA  GTAGTCAGAGCTAGAGCCCTGGGGGCTACCAGTCC |
| RXRA-99-3.1-F | GAGCTCGGATCCGCCACCATGCTCAATTCACCCATGAAC |
| RXRA-219-3.1-R | GTAGTCAGAGCTAGAGCCGTCCTTGCCCCGCTGCCGCT |
| HDAC5-1-3.1-F | GAGCTCGGATCCGCCACCATGAACTCTCCCAACGAG |
| HDAC5-1115-3.1-R | TGGGTAAGAGCTAGAGCCCAGGGCAGGCTCCTGCTC |
| HDAC5-292-3.1-R | TGGGTAAGAGCTAGAGCCGATCTCAACTGCTCTCTT |
| HDAC5-674-3.1-R | TGGGTAAGAGCTAGAGCCTGTGGTGAAGAGGTGCTT |
| HDAC5-293-3.1-F | GAGCTCGGATCCGCCACCATGACCGGCACGGGGCCTGGGGT |
| HDAC5-675-3.1-F | GAGCTCGGATCCGCCACCATGGGTGTGGTCTATGACACG |


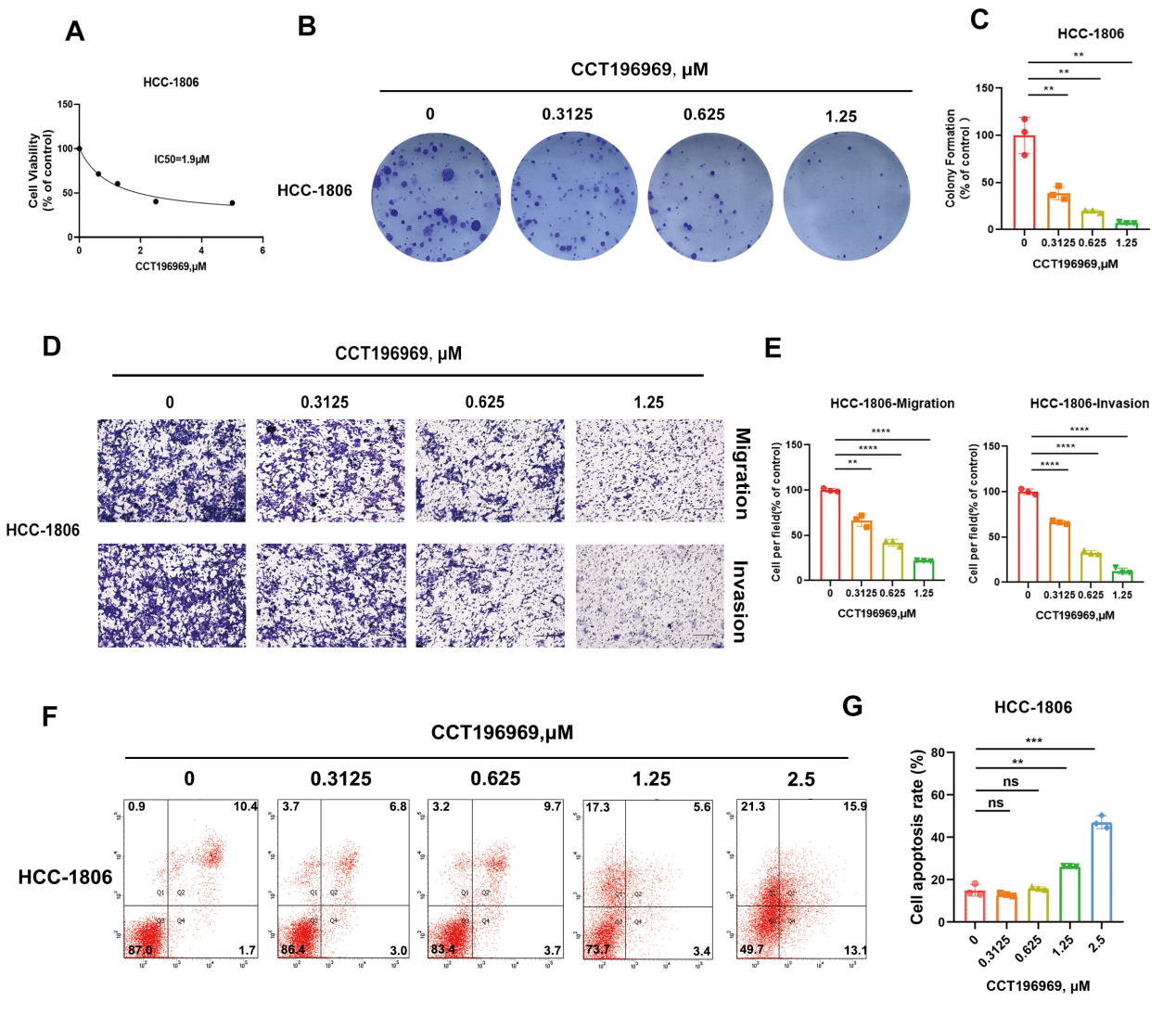


Supplementary Figure 1. CCT196969 significantly inhibits TNBC in vitro. A-G. HCC-1806 cells were treated with different concentrations of CCT196969 for 48 hours, and the following experiments were performed: A. CCK-8 experiment, with data from three repeated experiments. B-C. Clone formation experiment was repeated three times, the number of cell colonies was counted, and the effect of CCT196969 on cell growth was observed. Figure B is the representative result, and Figure C is the statistical figure. D-E. Cell invasion and migration experiment, three independent data were collected for statistical analysis. Figure D is the typical result, and Figure E is the statistical figure. Scale bars, 20 μm. F-G. Apoptosis experiment, Annexin V-FITC/PI double staining flow assay was used to collect three independent data for statistical analysis. Figure F represents the typical result and Figure G is the statistical graph. All values are mean ± SD. The statistical significance was determined by t test, *, *P*< 0.05; **, *P*< 0.01; ***, *P* < 0.001, *****P*<0.0001.


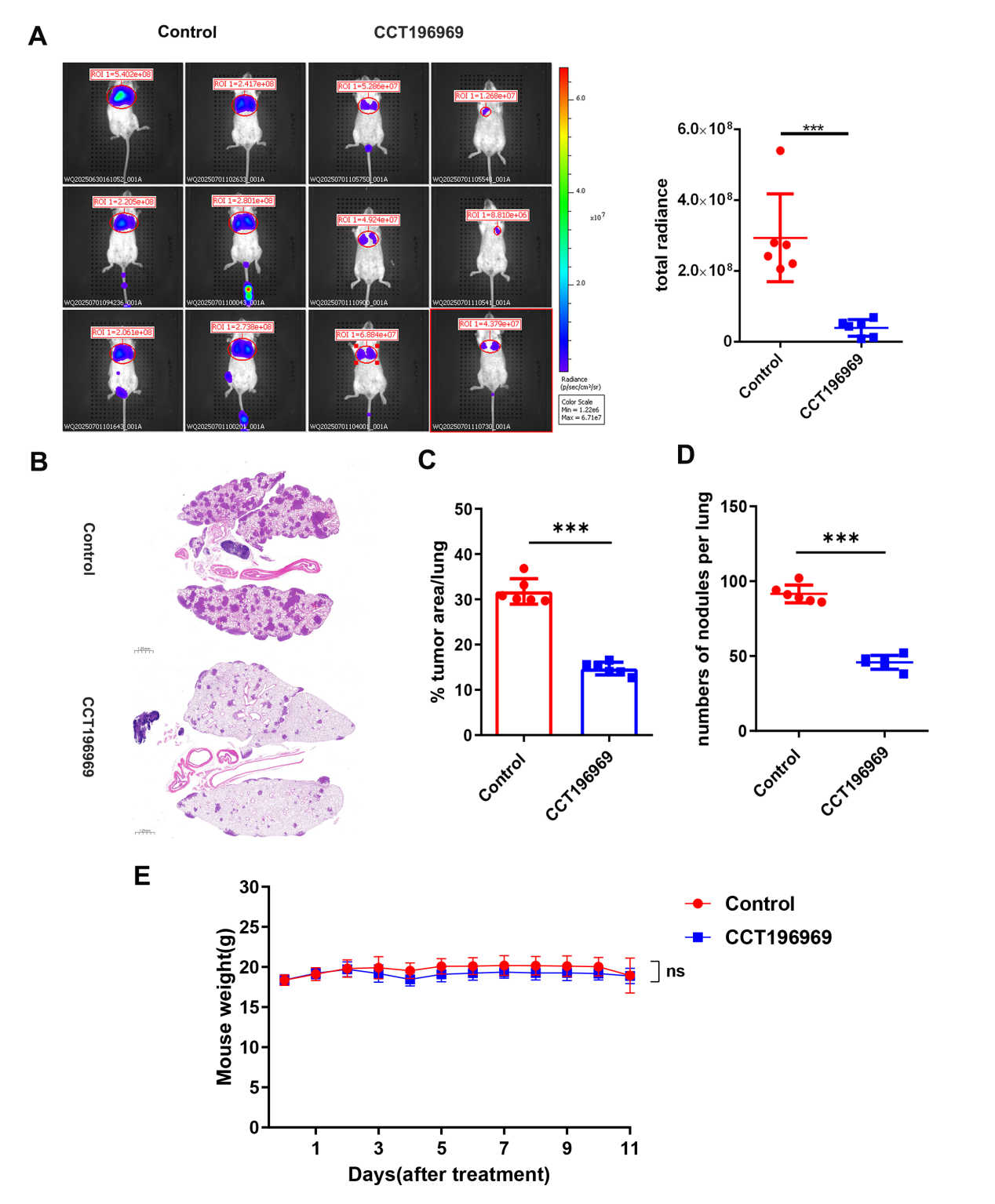


Supplementary Figure 2. CCT196969 inhibits the lung metastasis of TNBC in vivo. A. Balb/c nude mice were injected tail vein with 4T1-luc cells. After treating the mice with CCT196969 (10 mg/kg, gavage once a day) for 11 days, in vivo imaging of the mice was performed using the IVIS Spectrum system (representative graph on the left and statistical graph on the right). B. Representative images of lung metastatic nodules. C. The percentage of tumor area in lung tissue in Control group and CCT196969 treatment group. D. The number of nodules per lung in Control group and CCT196969 treatment group. E. Weight changes of mice in Control group and CCT196969 treatment group. All values are mean ± SD. The statistical significance was determined by t test, *, *P*< 0.05; **, *P*< 0.01; ***, *P* < 0.001.


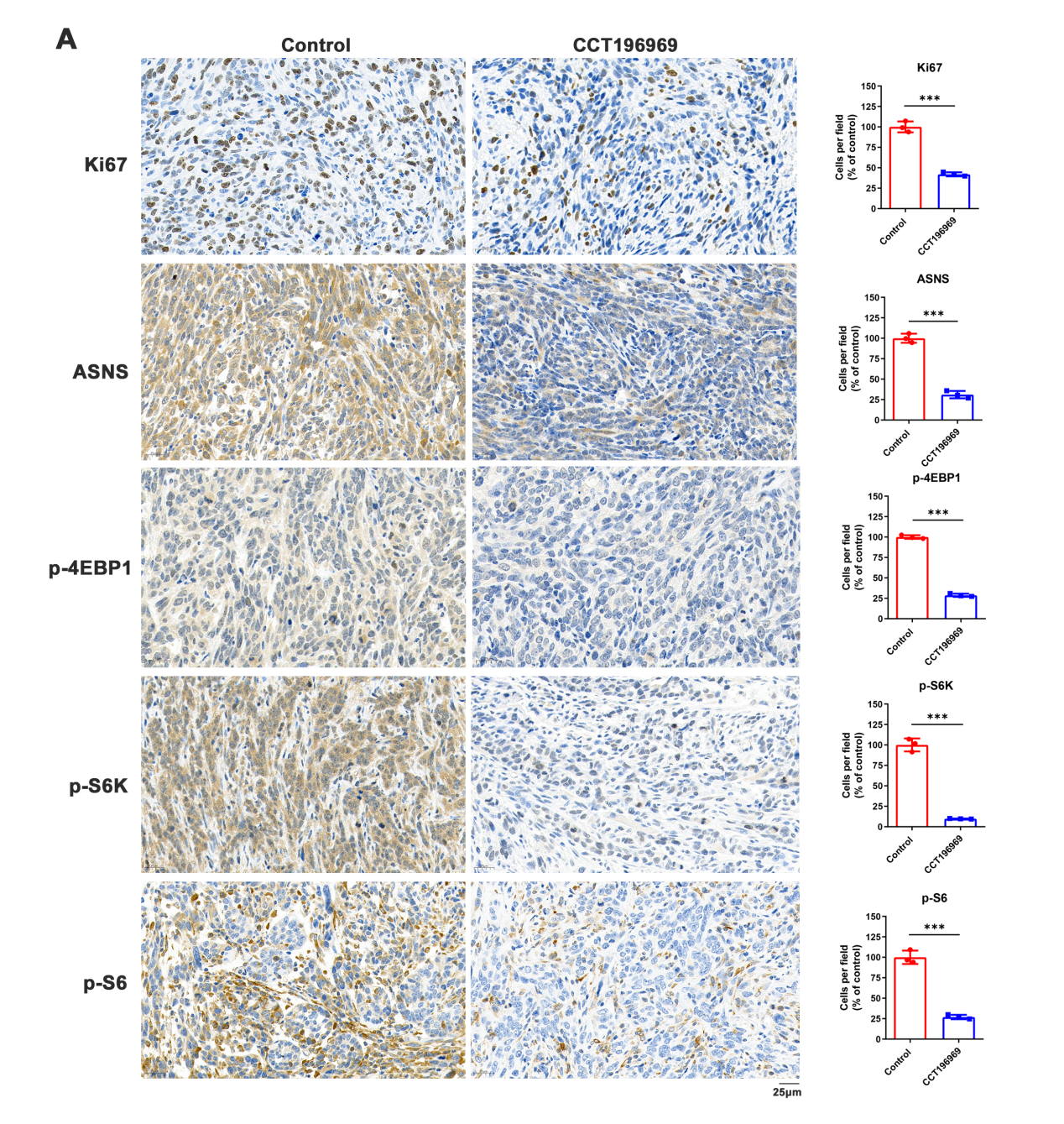


Supplementary Figure 3. CCT196969 inhibits the expression of ASNS and its downstream mTORC pathway as well as proliferation. A. Immunohistochemical evaluation of the expressions of ki67, ASNS, p-S6K, p-S6 and p-4EBP1 in orthotopic tumor tissues from Figure 1H. (Three tumor tissues were randomly selected for each group, representative graph on the left and statistical graph on the right). Scale bars, 25 μm. Data were mean ± SD, **P*<0.05, ***P*<0.01, ****P*<0.001.


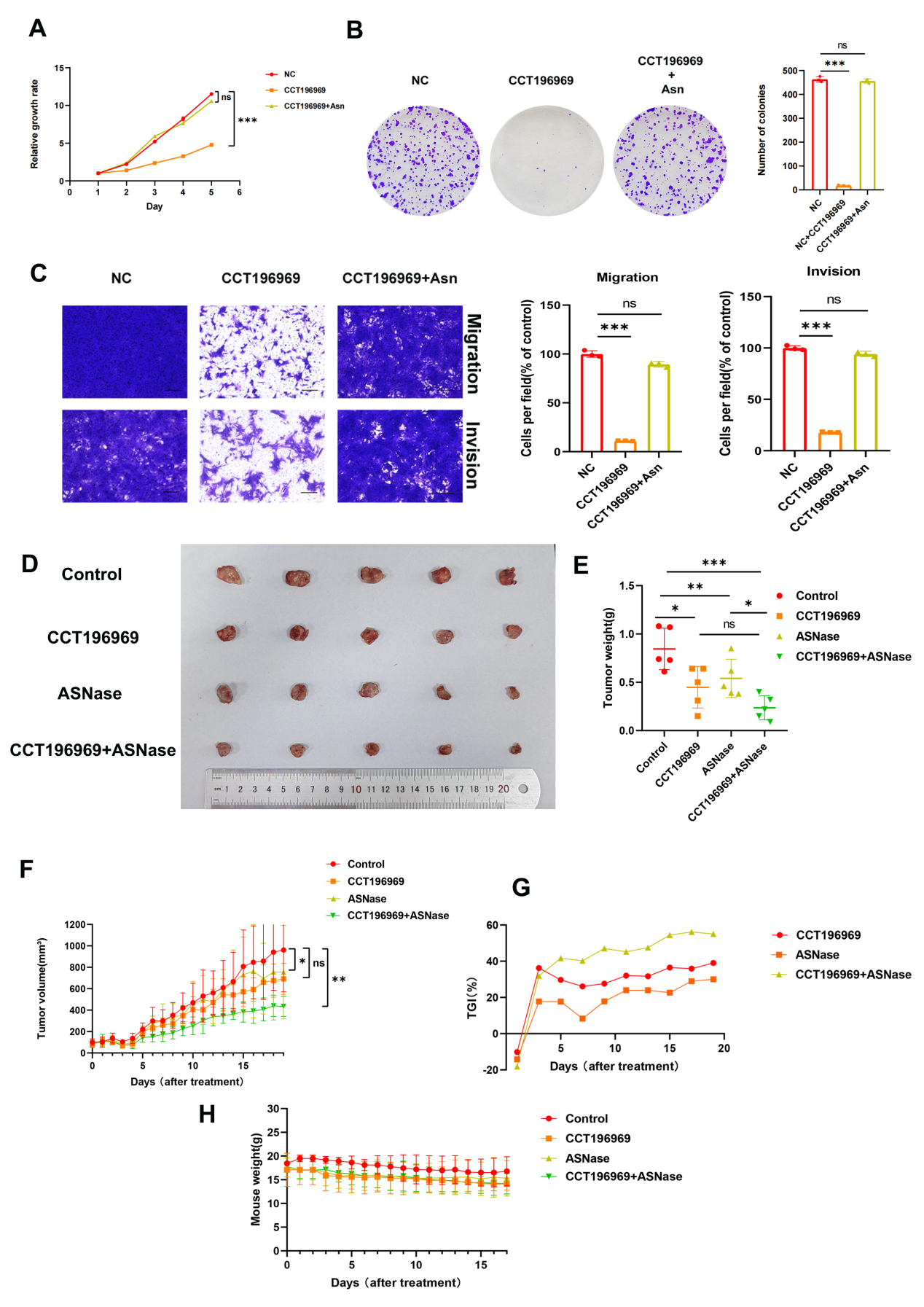


Supplementary Figure 4. CCT196969 combined with ASNase is more effective in suppressing TNBC. A-C. After 4T1 cells were treated with CCT196969 (0.625 μM) for 48 h and cultured with or without asparagine (1 mM) for 48 h, the following experiments were performed: A. CCK-8 experiment (3 independent experiments). B. Clone formation experiment (3 independent experiments, the left picture is the representative graph, the right picture is the statistical graph). C. Transwell experiment (3 independent experiments, representative graph on the left and statistical graph on the right). Scale bars, 20 μm. D-G. BALB/c mice were randomly divided into 4 groups, in situ injection of 4T1 cells, tumor formation was given CCT196969 (5 mg/kg, gavage), ASNase (40 UI, intraperitoneal injection) or combined administration, Control group was given normal saline: D. Comparison of tumor size in each group (n=5 in each group). E. tumor weight map. F. Tumor growth curve. G. Tumor growth inhibition (TGI) value. H. Map of weight change in mice. Data were mean ± SD, **P*<0.05, ***P*<0.01, ****P*<0.001.


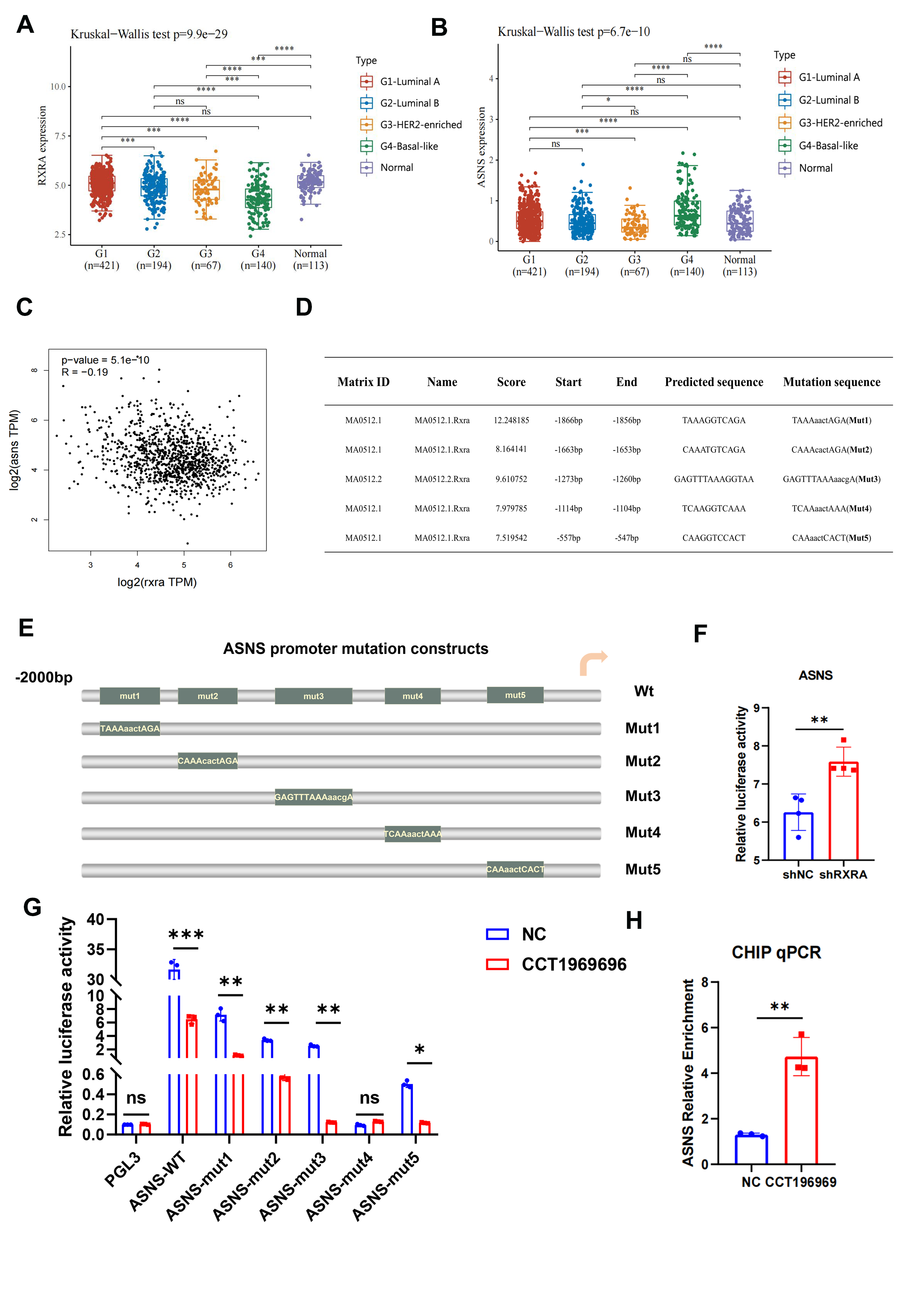


Supplementary Figure 5. RXRA inhibits transcription by binding to the -1114/-1104 region of the ASNS promoter. A. The expression of RXRA in breast cancer and normal tissues was analyzed by TCGA database. B. The expression of RXRA in different breast cancer subtypes was analyzed by TCGA database. C. TCGA database analysis of the correlation between RXRA and ASNS. D-E. JASPAR website predicted the binding sites of RXRA and ASNS promoter and mutation diagram was designed. F. Dual luciferase assay was used to detect the activity of ASNS promoter in shNC-4T1 and shRXRA-4T1 cells. G. Dual luciferase assay was used to detect the effect of CCT196969 treatment on promoter activity of 4T1 cells transfected with wild type and mutant ASNS promoter plasmids. H. CHIP-qPCR was used to detect the binding changes of RXRA to the -1114/-1104 region of the ASNS promoter before and after CCT196969 treatment. Data were mean ± SD, **P*<0.05, ***P*<0.01, ****P*<0.001, *****P*<0.0001.


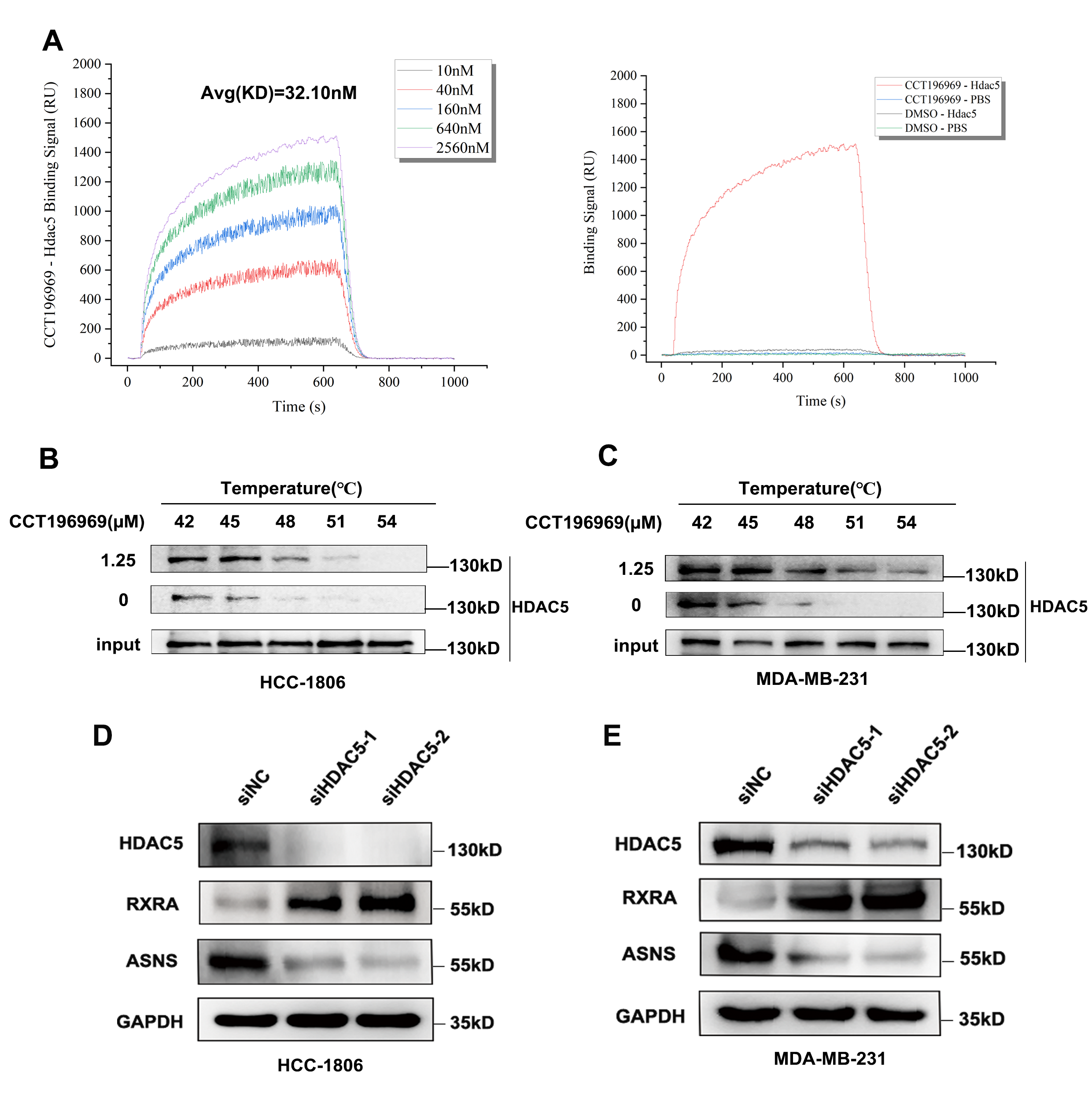


Supplementary Figure 6. HDAC5 is the direct target of CCT196969. A. Concentration gradient-specific binding curves of the interaction between different concentrations of HDAC5 and the stationary phase CCT196969. The interaction between HDAC5 protein and CCT196969 was assessed on the chip. Western blot was used to detect the thermostability of HDAC5 in HCC-1806 (B) and MDA-MB-231 cells (C) before and after CCT196969 treatment. The input cell lysate is not subjected to thermal treatment. Western blot was used to detect the protein expression of RXRA and ASNS after HDAC5 knockdown in HCC-1806 (D) and MDA-MB-231 cells (E).
